# The major secreted protein of the whipworm parasite tethers to matrix and inhibits interleukin-13 function

**DOI:** 10.1038/s41467-019-09996-z

**Published:** 2019-05-28

**Authors:** Allison J. Bancroft, Colin W. Levy, Thomas A. Jowitt, Kelly S. Hayes, Seona Thompson, Edward A. Mckenzie, Matthew D. Ball, Eamon Dubaissi, Aidan P. France, Bruno Bellina, Catherine Sharpe, Aleksandr Mironov, Sheila L. Brown, Peter C. Cook, Andrew S. MacDonald, David J. Thornton, Richard K. Grencis

**Affiliations:** 1 Lydia Becker Institute for Immunology and Inflammation, Manchester, M13 9PT UK; 2 Wellcome Trust Centre for Cell Matrix Research, Manchester, M13 9PT UK; 3 Division of Infection, Immunity and Respiratory Medicine, Manchester, M13 9PT UK; 4 School of Biological Sciences, Faculty of Biology, Medicine and Health, Manchester Academic Health Science Centre, University of Manchester, Manchester, M13 9PL UK; 5 Manchester Institute of Biotechnology, University of Manchester, 3.020 Garside Building, 131 Princess Street, Manchester, M1 7DN UK; 6 Manchester Collaborative Centre for Inflammation Research, Division of Infection, Immunity and Respiratory Medicine, School of Biological Sciences, Faculty of Biology, Medicine and Health, Manchester Academic Health Science Centre, Core Technology, University of Manchester, 46 Grafton Street, Manchester, M13 9NT UK

**Keywords:** Parasite host response, Parasite immune evasion, Structural biology

## Abstract

Infection by soil transmitted parasitic helminths, such as *Trichuris spp*, are ubiquitous in humans and animals but the mechanisms determining persistence of chronic infections are poorly understood. Here we show that p43, the single most abundant protein in *T. muris* excretions/secretions, is non-immunogenic during infection and has an unusual sequence and structure containing subdomain homology to thrombospondin type 1 and interleukin (IL)−13 receptor (R) α2. Binding of p43 to IL-13, the key effector cytokine responsible for *T. muris* expulsion, inhibits IL-13 function both in vitro and in vivo. Tethering of p43 to matrix proteoglycans presents a bound source of p43 to facilitate interaction with IL-13, which may underpin chronic intestinal infection. Our results suggest that exploiting the biology of p43 may open up new approaches to modulating IL-13 function and control of *Trichuris* infections.

## Abstract (web summary)

In the study, the authors identify a protein excreted by the parasite *Trichuris muris*, p43, which can modulate IL-13 function, a key cytokine involved in host protection. These data suggest that p43 may be a novel therapeutic target for both whipworm infections and IL13 mediated pathologies.

## Introduction

Soil-transmitted helminths (STHs) are responsible for infections that form part of the World Health Organization defined neglected tropical diseases, and as such present a considerable public health problem currently affecting around 1.9 billion people worldwide^1^. Infection with *Trichuris trichiura*, commonly known as whipworm (one of the four major STH), currently infects approximately 477 million people^1^. As a member of the prevalent *Trichuris* genus, these parasites occupy a distinct niche, the epithelial layer of the cecum, and proximal colon of the large intestine^2^, and parasites exist as long-lived, chronic infections. Little is known about how they maintain prolonged survival within the host, although there is remarkable genotypic and phenotypic similarity among *Trichuris* species^3,4^, regardless of the host, suggesting that common mechanisms underlying chronic infection may operate.

In addition to underpinning the role of interleukin-13 (IL-13)-mediated protective immunity to gastrointestinal-dwelling nematode infections,^5,6^ the naturally rodent-infecting species, *Trichuris muris*, has been successfully used as an experimental model of chronic STH helminth infection^7,8^. Under natural conditions, infection occurs by repeated low-level ingestion of eggs; IL-13-dependent protective immunity is slow to develop and partial at best. Parasite survival, therefore, ultimately depends upon the ability of *Trichuris* to disrupt effective IL-13-mediated immunity. Understanding how this group of parasites is able to achieve this, is vital to infection control, and potential elimination as current anthelmintic therapy in humans, is rarely completely effective, with *T. trichiura* often being the most persistent human STH following anthelmintic treatment^9,10^.

Here we characterize the structure and function of p43, the single most abundant protein in secretions from adult *T. muris*, whipworm. Structural determination by X-ray crystallography reveals potential interaction with glycosaminoglycans and the host cytokine IL-13. Binding studies confirm high-affinity binding to heparan sulfate (HS) and IL-13. Moreover, p43 inhibits IL-13 immune function both in vitro and in vivo. The presence of p43 homologs in closely related whipworms, such as *T. trichiura,* is suggestive of common immunomodulatory function.

## Results

### p43 location in the parasite and the host

**Fig. 1:**
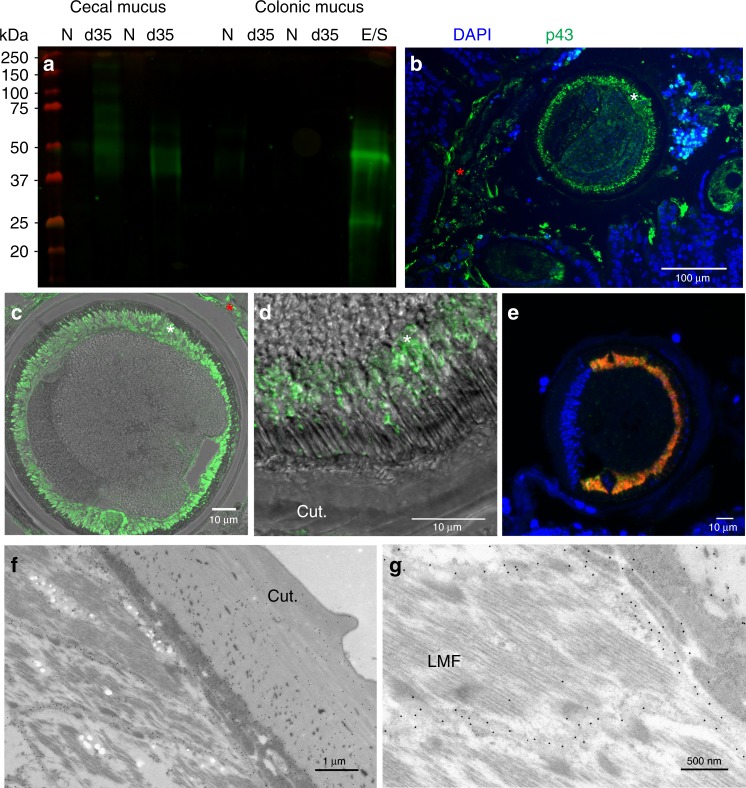
p43 is associated with the longitudinal muscle of the parasite and the intestinal mucus and the surrounding epithelial matrix of the host. **a** Western blot of cecal and colonic mucus samples from naive (N), *Trichuris muris-*infected (d35) mice, and *T. muris* whole excretome/secretome (E/S) probed with anti-p43 antibody. **b** Cecal sections from infected mice stained with anti-p43 and 4′,6-diamidino-2-phenylindole, dihydrochloride (DAPI) (merged), demonstrating staining within the worm (white asterisk) and extracellularly within the host intestinal lumen (red asterisk). **c**, **d** Transverse section of a worm taken from cecal sections from infected mice stained with anti-p43. **d** p43 staining within the worm (merged with bright field). **e** Transverse section of an adult *T. muris* hybridized with a p43 Cy5 probe (red) and myosin fluorescein isothiocyanate (FITC) probe (green); a merged image is shown, counterstained with DAPI. **f**, **g** Electron micrograph of adult *T. muris* stained with anti-p43 and colloidal gold particle-conjugated secondary antibodies. (Cut.= cuticle; LMF = longitudinal muscle fibers; white asterisk = within the worm; red asterisk = within the host intestinal lumen)

The excretome/secretome (E/S) of parasitic nematodes is extensive and represents a major source of extracellular parasite material that can interact with the host, containing both immunogens and immunomodulatory molecules^11^. The E/S of adult *T. muris* is no less extensive^12^ but is dominated by a single protein that can be visualized by sodium dodecyl sulfate-polyacrylamide gel electrophoresis (SDS-PAGE) resolving at a band of approximately 43 kDa and was termed p43 (Supplementary Fig. 1a). Multi-angle light scattering of native *T. muris* E/S confirmed the dominance of p43 in E/S as a monomeric protein (Supplementary Fig. 1b), which is encoded for by TMUE_3000012139, a poly-cysteine and histidine-tailed protein gene (Supplementary Fig. 2a, b)^3^. The protein encoded for by this gene has an unknown function, but is the tenth most highly expressed in adult worms^3^, suggesting major investment by the parasite and thus importance in the host/parasite relationship. Real-time polymerase chain reaction (qPCR) confirmed p43 RNA expression in all life cycle stages (Supplementary Fig. 1c). Secretion of p43 was confirmed by culturing adult worms in increasing concentrations of sodium azide (Supplementary Fig. 1d). Western blotting (Fig. 1a) and mass spectrometry (Supplementary Table 1) confirmed the presence of p43 in the secreted mucus from chronically infected mice. It is clearly present in the mucus from the cecum, the parasite niche as opposed to the colon, where parasites are not present after low-dose chronic infection (Fig. 1a, Supplementary Table 1). Accordingly, p43 protein can be readily observed in both the epithelial matrix surrounding the parasite in situ and in the host intestinal lumen (Fig. 1b–d, Supplementary Fig. 3a). The p43 protein is located beneath the cuticle in adult parasites (Fig. 1c, d) and transcription of the p43 gene and myosin gene is co-located (Fig. 1e, Supplementary Fig. 3b), suggesting muscle origin, and is consistent with the protein residing within or alongside the longitudinal muscle layer of the parasite (Fig. 1f, g). Immunogold electon microscopy confirmed labeling of p43 protein between the muscle cells (Fig. 1f, g, Supplementary Fig. 3c). Taken together with the considerable change in size from L1 to adult (approximately 5000-fold increase in body/muscle volume over a 35-day period), it is clear that adult parasites produce the greatest quantity of p43 of all life cycle stages.

### Crystal structure of p43

Sequence-based similarity searches reveal high levels of orthology between p43 and predicted proteins identified in other *Trichuris* species and closely related *Trichinella* species, suggesting that the molecules may be isostructural and therefore perform a common function within this group of parasites (Supplementary Fig. 4). *Trichuris muris* p43 comprises 397 amino acids (AA), 36 cysteine residues (9%), and a histidine-rich C-terminal region (45% of terminal 29 residues are His). Analyses of the primary sequence reveal an internal repeat with residues 1–180 and 181–394 sharing 34% amino acid sequence identity. To better understand the possible function of p43, we determined the three-dimensional structure at 1.8 Å using X-ray crystallography, Protein Data Bank (PDB) ID 6QIX.

**Fig. 2:**
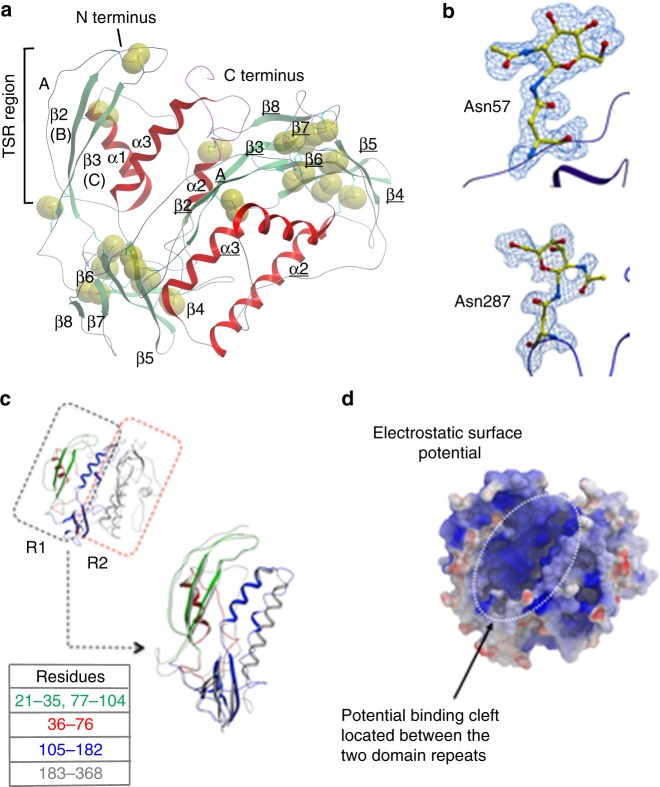
Crystal structure of p43 showing glycosylation sites, thrombospondin type one domain (TSR-1), and potential binding cleft. **a** A ribbon representation of p43 crystal structure; helices are colored red and β-strands green. Individual helices and strands are labeled α1, β1 for the first domain repeat and α1, β1 for the second domain repeat. The three characteristic strands of a TSR-1 are labeled A, B, and C, respectively. The sulfur atoms are shown as Corey–Pauling–Koltun (CPK) spheres highlighting disulfide bonds. **b** Residues 57 and 287 are shown in an all-atom representation colored by atom type along with their associated *N*-acetylglucosamine glycosylation. The electron density depicted is a feature-enhanced map (FEM) contoured at 1 sigma. Cartoon representation of the protein backbone is shown in blue. **c** Ribbon representation of p43 structure highlighting the domain repeat observed along with residues involved in the TSR-1 region (21–35, 77–104 in green), the α1 and flexible linker region (36–76 in red), and the highly disulfide linked β- sandwich region extending into α2 and α3 (105–182 in blue) forming the core of the protein. The second domain repeat is shown in ribbon representation (183–368 in gray). The two domain repeats have been superimposed based upon C-α positions. **d** Surface representation of p43 colored by electrostatic surface potential calculated using the Rapid-Exact-Boundary Element (REBEL) method as implemented in ICM-PRO (blue +ve, white neutral, and red −ve). A potential binding cleft located between the two adjacent domain repeats is highlighted

The protein has mixed α and β composition with 16 β-strands and 6 α-helices (Fig. 2a). The crystal structure reveals contiguous electron density for residues 21–368, and no electron density is observed for the histidine-rich C-terminal region. The structure shows that all 36 of the available cysteine residues are involved in the formation of 18 intramolecular disulfide bonds (Supplementary Fig. 5a). In each of the two domain repeats, eight disulfides are conserved, while the bond between C274 and C277 has no equivalent in domain 1 and C131 from domain 1 bonds to C362 of domain 2. The disulfide bonding pattern is unevenly distributed throughout the protein clustering around the β-sandwich region (β4–β8, Fig. 2, Supplementary Fig. 5a). There are three potential N-glycosylation sites predicted at positions 57, 174, and 287, and the crystal structure shows clear evidence of glycosylation at residues 57 and 287, but not at position 174 (Fig. 2b). In addition to these three N-glycosylation sites, there is a potential site of C-mannosylation with p43 containing the W-x-x-W motif associated with such a modification^13^.

Structure-based similarity searches of the PDB reveal no hits in the Dali server^14^, indicating unique overall structure, while ProBis (Protein Binding Sites), a web server for detecting structurally similar binding sites in proteins^15^, highlights residues (21–25 and 77–104) as having significant similarity to thrombospondin type 1 repeat protein (TSP-1) displaying the characteristic antiparallel three-stranded β-sheet with a disrupted A strand (Fig. 2c, Supplementary Fig. 5b). The two top hits from ProBis were for micronemal protein 2 from *Toxoplasma gondii*, an adhesin involved in parasite binding to a matrix prior to cell invasion^16,17^, and human IL-13R α2, a regulatory receptor that controls IL-13 activity^18^. The regions of similarity for each are restricted to specific residues residing within the TSP-1 domains, providing intruiging possibilities for potential roles of p43. Surface representation of p43 by electrostatic surface potential indicates a negatively charged region, suggestive of a possible binding cleft between the two domain repeats (Fig. 2d).

### p43 binds to IL-13 and glycosaminoglycans

**Fig. 3:**
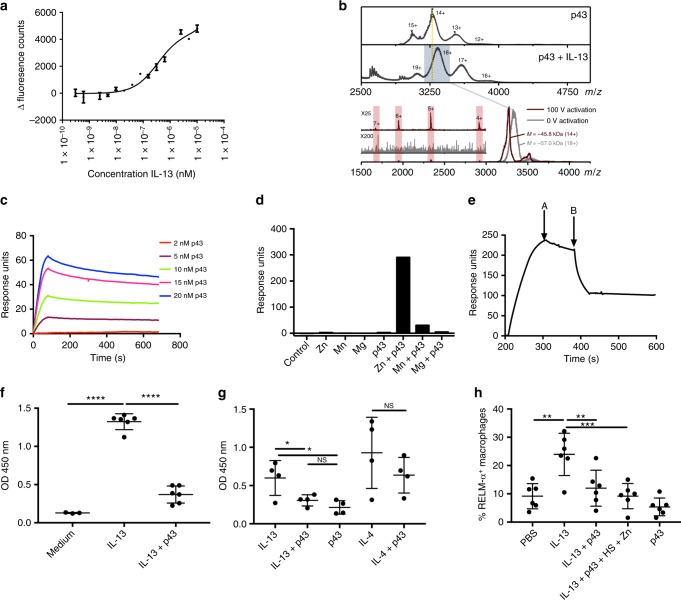
p43 binds to interleukin-13 (IL-13) and the extracellular matrix in vitro. In vivo, p43 downregulates IL-13-induced inflammation. **a** Microscale thermophoresis (MST) of IL-13 binding to NHS-647-labeled p43. The results are the mean of three separate analyses ±standard deviation. **b** Mass spectrum of p43 presenting with a narrow charge state distribution (12–15^+^) of heterogenous peaks centering on the 14^+^ charge state, upper panel. Mass spectrum of the p43:IL-13 complex is shifted to higher masses, middle panel. Collision-induced dissociation (CID) experiments were carried out on the mass-selected 18^+^ charge state of the p43:IL-13 complex. Under “soft” tuning conditions (gray trace), the 18^+^ charge state gave a broad peak (*m*/*z* of 3328). At a trap collision energy of 100 V, which promotes gas-phase protein activation (red trace), the p43:IL-13 mass selected peak shifts to a lower *m*/*z* (*m*/*z* 3260). The associated loss of mass of the p43:IL-13 18^+^ ion corresponds to the dissociation of a single IL-13 molecule (11.7 kDa). Inset peaks corresponding to the 4–7^+^ charge states of IL-13 at a collision energy of 100 V, which were not present at 0 V, lower panels. **c** Surface plasmon resonance (SPR) of biotinylated heparan sulfate (HS) and increasing amounts of p43. **d** SPR showing zinc-dependent binding of biotinylated HS with p43. **e** SPR of biotinylated HS binding to p43 with IL-13 competition (A, p43 injection stops B, IL-13 injected). All in vitro experiments from **a** to **e** were repeated at least 3 times and a representative experiment has been shown. **f**, **g** Resistin-like molecule-α (RELM-α) produced by peritoneal exudate cells (PECs) after in vitro stimulation by IL-13 ± p43 or IL-4 ± p43 measured by enzyme-linked immunosorbent assay (ELISA). **f** Six technical replicates from two pooled mice, *p* = 204.6, DF = 12, ****p < 0.0001. **g** RELM-α from four individual mice. Analysis by one-way analysis of variance (ANOVA), *p* = 7.422, DF = 9, IL-13 vs. IL-13  ± p43, *p = 0.0489. **h** Percentage of RELM-α-positive interstitial lung macrophages from six individual C57BL/6 mice given administration of IL-13 ± p43 to the respiratory tract. Data are from two experiments, *n* = 6 individual C57BL/6 mice. Bars denote means ± SEM. Analysis by one-way ANOVA; the data passed the Shapiro–Wilk and KS normality tests, *p* = 10.38, DF = 25, IL-13 vs. IL-13 ± p43, **p = 0.0063, IL-13 vs. IL-13 ± p43 + HS + Zn, ***p = 0.0007

As protective immunity to *T. muris* is dependent on IL-13 function in the intestine, we reasoned that p43 may bind IL-13 and serve as an immunomodulatory molecule. In silico docking of IL-13 binding to p43 is shown in Supplementary Fig. 6. p43 was shown to bind IL-13 directly by microscale thermophoresis (MST) with an equilibrium-binding constant *K*_D_ in the order of 420 ± 120 nM (Fig. 3a). Native mass spectrometry and collision-induced dissociation (CID) experiments showed p43-bound IL-13 with a 1:1 stoichiometry (Fig. 3b) and confirmed that p43 was a flexible molecule with the potential for conformational change upon activation (Supplementary Fig. 7). As predicted by thrombospondin type one domain (TSR-1) homology, p43 was also shown to bind readily to glycosoaminoglycans via enzyme-linked immunosorbent assay (ELISA) (Supplementary Fig. 8) and this was confirmed for HS via surface plasmon resonance (SPR), with an affinity in the region of 10 nM (Fig. 3c). Moreover, p43/HS binding was highly dependent upon the presence of zinc (Fig. 3d). Addition of IL-13 to p43 bound to HS using a competitive SPR method, uncoupled the p43 from HS (Fig. 3e), raising the possibility that the extracellular matrix may present p43 in a manner receptive to subsequent binding to IL-13. Taken together, the data indicate that p43 may interfere with the biological function of IL-13. To test this in vitro, peritoneally derived exudate cells (PECs) were incubated with IL-13 to stimulate the production of resistin-like molecule-α (RELM-α) in the presence or absence of p43. p43 significantly inhibited the induction of RELM-α induced by IL-13 but not IL-4 (Fig. 3f, g), showing that p43 inhibited IL-13 function. p43 was also shown in vivo to significantly inhibit the induction of RELM-α^+^ lung interstitial macrophages^19–22^ following administration of IL-13 to the respiratory tract (Fig. 3h, Supplementary Fig. 10).

### Function of IL-13 in vitro and in vivo

**Fig. 4:**
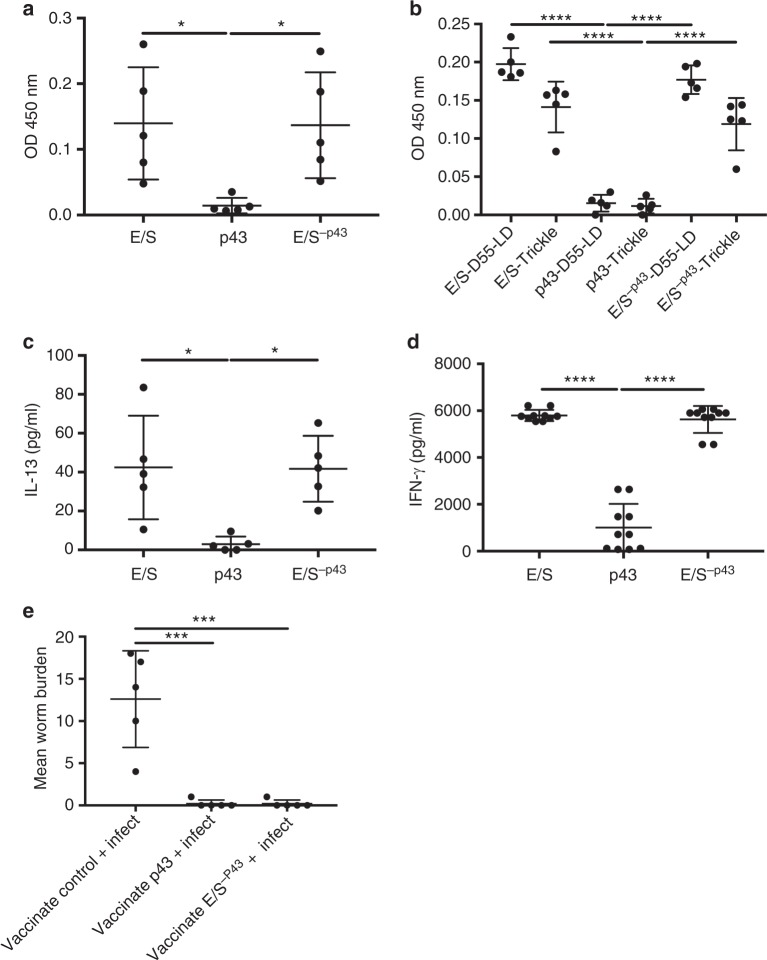
There is a lack of host immune reactivity to p43, although p43 is successfully able to vaccinate mice against infection. **a**, **b** Anti-mouse Ig (G, M, and A) enzyme-linked immunosorbent assay (ELISA) using serum from infected C57BL/6 mice. **a** Day 35 post infection (p.i.) serum from mice given a high-dose *T. muris* infection. **b** Serum from C57BL/6 mice 55 days p.i. earlier (E/S-D55-LD) or repeated low-dose (trickle) *T. muris* infections (day 0, 2, 4, 7, 9, and 11) at day 35 p.i. ELISA plates were coated with whole *T. muris* E/S, native p43, or E/S−^p43^, and serum was diluted at 1/80. **c** Interleukin-13 (IL-13) cytokine production from mesenteric lymph node cells (MLNCs) from mice infected with 200 *T. muris* eggs and culled at day 21 p.i. **d** Interferon-γ (IFN-γ) production from MLNCs from mice infected with 20 *T. muris* eggs and culled at day 21 p.i. In both assays, cells were restimulated with whole E/S, native p43, or ES^−p43^. **e** Worm burdens of mice at 35 days p.i. Mice were vaccinated with 50 μg of p43, or E/S^−p43^ subcutaneously with alum or with alum alone (vaccination control) on day −28 and day −14 p.i. and infected at day 0 with 20 *T. muris* eggs. Data in **a**–**e** have been repeated at least 3 times and *n* = 5. Data from noninfected controls were below the detectable limits of the assay for data shown in **a**–**d**. All data are presented as mean ± SEM. A one-way analysis of variance (ANOVA) was used to analyze the data, **a** *F* value = 5.494, DF = 12, E/S vs. p43, *p = 0.0330, p43 vs. E/S^−p43^, *p = 0.0370; **b** *F* = 58.25, DF = 24, E/S-D55-LD vs. p43-LD-D55; E/S-Trickle vs. p43-Trickle; p43-LD-D55 vs. E/S^−p43^-LD-D55 and p43-Trickle vs. E/S-p43-Trickle, all ****p < 0.0001; **c** *F* = 7.577, DF = 12, E/S vs. p43, *p = 0.0135 and p43 vs. E/S^−p43^, *p = 0.0150; **d** *F* = 156.8, DF = 27, E/S vs. p43 and p43 vs. E/S^−p43^, ****p < 0.0001; **e** *F* = 24.27, DF = 12, vaccinated control + infected vs. vaccinated p43 and vaccinated control + infected vs. vaccinated E/S^−p43^, ***p = 0.0002

Previous work has shown that infected mice that are resistant (expel a high-dose infection) or susceptible (harbor a low-dose chronic infection) to *T. muris* infections make robust *T. muris* E/S-specific CD4+ T-helper (Th) cell recall responses and serum antibody responses^5^. To assess the contribution and importance of p43 to these responses, E/S in which p43 had been removed (E/S^−p43^), or purified native p43, were investigated by ELISA and in vitro cytokine production following acute or chronic infection. Strikingly, there was an absence of host immune response to p43 in comparison with both whole E/S and E/S^−p43^. Figure 4a, b demonstrated that the peripheral antibody responses in mice given an acute infection, a chronic infection for 55 days, or had received repeated low-dose infections (i.e., a trickle infection), were against the minor component antigens in the E/S, E/S^−p43^, and not against p43. Similarly, in vitro recall responses of mesenteric lymph node cells (MLNCs) from *T. muris*-infected mice given either a high-dose (200 eggs, characterized by IL-13 production) or a low-dose infection (20 eggs, characterized by interferon-γ (IFN-γ) production) showed similar cytokine profiles after re-stimulation with E/S and E/S^−p43^, whereas p43 stimulation resulted in negligible cytokine secretion, confirming lack of CD4+ T-cell priming against p43 during infection (Fig. 4c, d). To assess the capacity of p43 to be processed and activate antigen-presenting cells, bone marrow-derived dendritic cells (BMDDCs) were pulsed with fluorescently labeled p43, showing that p43 can localize alongside H2-M within the endosomal processing compartment (Supplementary Fig. 9a). Moreover, BMDDCs showed a modest dose-dependent increase in major histocompatibility complex II (MHC II) and CD86 expression following pulsing with p43, demonstrating that p43 can activate DCs (at least in vitro) similar to other archetypal helminth antigens, such as schistosomal egg antigen (SEA^23^; Supplementary Fig. 9b, c). However, unlike SEA, p43 does not induce downregulation of MHC II and CD86 after lipopolysaccharide stimulation (Supplementary Fig. 9d, e). To test the immunogenicity of p43 in vivo, mice were immunized subcutaneously with p43 (together with alum) and compared to immunization with E/S^−p43^. Vaccination with p43 protected mice against a challenge infection as effectively as E/S^−p43^ (Fig. 4e), indicating that p43 is immunogenic per se and can induce protection against infection, suggesting that immune responses targeting p43 are detrimental to parasite survival. Thus, despite a lack of immunogenicity during natural infection, p43 can function as a cryptic protective antigen.

## Discussion

Taken together, the data suggest that the large quantities of p43 secreted by adult parasites during chronic *T. muris* infection may serve to interfere with the development of IL-13-dependent host protective immunity. Previous work in other helminth systems has demonstrated the potential of parasite-derived molecules in modulating immune responses either directly or indirectly primarily by influencing the initiation of immune responses or generation of regulatory cell populations^24–26^. In contrast, this work shows immunomodulation at the effector stage of the immune response, and we hypothesize that the unique structural features of p43 may reflect this ability, although further work is required to explore other potential cytokine ligands that may be present in the parasite niche. The composition and structure of p43 are consistent with a molecule that would tether to the extracellular matrix-rich environment into which it is released and be able to survive the harsh conditions that it would encounter in the microbially abundant ecosystem of the cecum and colon. Tethering to matrix molecules such as HS may also contribute to its lack of immunogenicity during infection^27^. It would be interesting to further investigate the binding repertoire of p43 going forward. The fact that homologs of p43 in other trichuroid nematodes, such as the human parasite *T. trichiura* and *T. spiralis*^28^ are likely to be isostructural (Supplementary Fig. 4) strongly supports the hypothesis that they may play a similar role in their respective infections, particularly related to the specific intestinal epithelial niche that they all occupy. Whether p43 and “p43-like” molecules have other novel functions operating within the parasite itself or the host during infection, remains to be discovered and will require further investigation. In summary, we identify p43 as a prototype of *Trichuris*-derived immunomodulatory molecule with a unique structural composition that may serve as a key target for intervention in whipworm infection in man or animals by both vaccination and novel drug-based therapy. Moreover, it offers potential to inform on the development of novel cytokine inhibition strategies, with IL-13 being a critical mediator of the allergic response at mucosal surfaces.

## Methods

### Parasites

The Edinburgh strain of *T. muris* was used in all experiments originally obtained from The Wellcome Research Laboratories, London^29^ and is routinely passaged within our laboratory.

### Animals

Male animals were used unless specifically stated at 6–8 weeks of age and were housed for 7 days prior to experimentation. C57BL/6 and BALB/c mice were purchased from Envigo (Huntingdon, UK) or Charles River (UK) and severe combined immunodeficient (SCID) mice were bred in-house at the Biological Services Facility (BSF) at the University of Manchester. Experiments were performed under the regulations of the Home Office Scientific Procedures Act (1986) and were subject to local ethical review by the University of Manchester Animal Welfare and Ethical Review Body (AWERB) and followed ARRIVE guidelines. The mice utilized in these experiments were not randomized, but cages were randomly assigned to different treatment groups for vaccination experiments.

### E/S material

SCID mice were infected with 200 *T. muris* eggs by oral gavage. Forty two days later, mice were killed using CO_2_, and the cecum and proximal colon were removed. The cecum was split and washed in RPMI-1640 plus 500 U/ml penicillin and 500 μg/ml streptomycin (all from Sigma-Aldrich, UK). Worms were removed using fine forceps and cultured for 4 h in RPMI-1640 plus penicillin/streptomycin as above at 37 °C. The E/S from worm culture was centrifuged to pellet the eggs and filtered through a syringe filter of 0.22 μm (Sartorius, Gottingen, Germany). After concentration using centrifugal filter units (Merck™Amicon, Fisher Scientific, UK) and dialysis against phosphate-buffered saline (PBS), the concentration of the E/S was determined using a Nanodrop (Labtech International, Heathfield, UK). It was aliquoted and stored at −20 °C until use.

### Western blot

Mucus samples extracted from *T. muris* infected or naive animals were analyzed on 4–12% (w/v) RunBlue™ SDS precise gels (Expedeon Ltd, Cambridgeshire, UK). Samples were prepared using RunBlue rapid SDS running buffer (Expedeon Ltd, Cambridgeshire, UK) and reduced in 50 mM dithiothreitol (Sigma-Aldrich, UK) for 10 min at 95 °C. Precision Plus Protein All Blue Standards (Bio-Rad Laboratories, Hertfordshire, UK) were used as molecular weight markers. Gels were run in XCell II Mini-cell electrophoresis tanks (Novex, San Diego, CA, USA) at 200 V for 60 min. Following electrophoretic separation, gels were analyzed for the presence of *T. muris* p43 in the mucus extract. The proteins were transferred onto a nitrocellulose membrane using an XCell IITM semi-wet Blot Module (Invitrogen, Thermo Fisher Scientific, UK) and run at 60 V for 90 min in 20% methanol in NuPAGE transfer buffer (Novex, San Diego, CA, USA). The membrane was briefly washed in Tris (hydroxymethyl amino methane)-buffered saline (TBS), polyoxyethylene sorbitan monolaurate (Tween-20) (Sigma-Aldrich, UK), 0.05% TBS Tween-20, and blocked in 5% nonfat milk (Marvel, UK) overnight at 4 °C. It was probed with polyclonal rabbit anti-p43 (raised against the native peptide sequence, Cambridge Research Biochemicals, Billingham, UK) and diluted 1/1000 in 5% nonfat milk for 90 min at room temperature. The membrane was washed in TBS Tween-20 5 times for 5 min, and then incubated for an hour with 800-nm goat anti-rabbit (926-32211, LI-COR, NE, USA) diluted 1/25,000 in 1% TBS Tween-20, and subsequently washed in 1% TBS Tween-20 4 times for 5 min with a final wash step in PBS. Blots were scanned on the LI-COR Odyssey CLx Infrared Imaging System (Li-COR Biotechnology-UK Ltd, Cambridge, UK).

### Secreted mucus extraction

Mouse intestines were removed and placed in a 12-well plate (Corning, NY, USA) on ice. The cecum and colon were separated and the fecal matter gently removed. The colon and cecum were cleaned by flushing the lumen with 2 ml of PBS using a blunt needle tip and a 2.5-ml syringe. The flow-through was collected and passed through the tissue again and retained in a 14-ml Falcon tube (Corning, NY, USA). Following PBS washes, the colons were flushed 5 times with 2 ml of 2 M urea (Melford, Ipswich, UK), and the flow-through was kept in a fresh clean tube. The cecal tissues were opened longitudinally and placed in 2 ml of 2 M urea and gently rotated for 1 min. Before and after mucus extraction, gut tissue snips were taken for histological analysis to ascertain whether any damage had occurred to the epithelium and if crypts were intact. The flow-through of PBS and 2 M urea flushes of each mouse were kept at 4 °C overnight and then concentrated using Sartorius Vivaspin 3-kDa molecular weight cutoff (MWCO) columns (Gottingen, Germany) spinning for 10 min at 19,000 × *g*. The solvent was exchanged to 6 M urea and all samples were at a volume of 200 μl. The mucus extracts were subjected to tandem mass spectrometry and SDS-PAGE analysis.

### Immunostaining

Five-micrometer sections were cut from the cecum of mice infected with 200 eggs of *T. muris*. After overnight drying at 42 °C, the sections were incubated in citroclear (TCS Biosciences, Buckingham, UK) for 5 min and then incubated in 100% ethanol and a gradient of 90%, 70%, and 50% ethanol for 2 min, followed by distilled H_2_O for 2 min. Sections were incubated in PBS for 5 min. The sections were blocked with 10% goat serum (Sigma-Aldrich, UK) for 30 min. The anti-p43-kDa antibody (Cambridge Research Biochemicals, Billingham, UK) was used at 1/400 in 1/10 blocking buffer overnight at 4 °C. The next day after washing in PBS, sections were incubated in goat anti-rabbit Alexa Fluor 488 (A-11034, Invitrogen, Thermo Fisher Scientific, UK) 1/800 in PBS for 1 h. Further washing in PBS was followed by incubation in 0.1% Sudan Black (Sigma-Aldrich, UK) in 70% ethanol in the dark. After a final wash in PBS, slides were allowed to air-dry and mounted in Mowiol^®^4-88 (Sigma-Aldrich, UK) mounting medium plus or minus 4′,6-diamidino-2-phenylindole, dihydrochloride (DAPI) (Sigma-Aldrich, UK). Images were collected on a Leica TCS SP5 AOBS inverted confocal microscope (Leica Microsystems, Milton Keynes, UK) using a ×20/0.50 Plan Fluotar objective and ×3 confocal zoom. The confocal settings were as follows: pinhole (1 airy unit), scan speed (1000 Hz unidirectional), and format (1024 × 1024). Images were collected using (hybrid) detectors with the following detection mirror settings: fluorescein isothiocyanate (FITC) 494–530 nm, using the white light laser with 488 nm (20%). When acquiring 3D optical stacks, the confocal software was used to determine the optimal number of Z sections. Only the maximum intensity projections of these 3D stacks are shown in the results. A Coolsnap ES2 camera (Photometrics, AZ, USA) was used to capture the images using Metavue v 7.8.4.0 software (Molecular Devices, CA, USA). Images were then processed and analyzed using Fiji software. FISH-infected mice were killed and their ceca removed. They were cut longitudinally and cecal contents removed in PBS. The cecum and proximal colon were cut into small sections and incubated in 4% paraformaldehyde for 60 min at room temperature. The sections were then washed in PBS and transferred to 70% ethanol and stored at −80 °C until use. Tissue processing was carried out using an automated tissue processor, Shandon Citadel 2000 (Thermo Scientific, UK), and the sections were embedded in paraffin wax. Five-micrometer sections were cut using a Leica RM 2235 microtome (Leica Biosystems, Milton Keynes, UK).

### Fluorescent in situ hybridization

For generation of the RNA probes, messenger RNA (mRNA) was extracted from adult worms by maceration in Trizol (Life Technologies, Thermo Fisher Scientific, UK) with a FastPrep and lysing matrix D (MP Biomedical, Fisher Scientific, UK). The p43 cDNA template to generate RNA probes was obtained by reverse transcriptase PCR from extracted mRNA using Superscript III (Invitrogen, Thermo Fisher Scientific, UK), followed by PCR amplification of p43 using specifically designed primers and Phusion DNA polymerase (NEB, UK). The following primers were used for p43: forward, 5′-GCAATGCTTCACTCGACCCG-3′ and reverse, 5′-TTGTGGTGCTCCTCTCCGTG-3′. For myosin, the following primers were designed from WormBase ParaSite^30^ using the *T. muris* genome and a paralog sequence of myosin 3 from *Caenorhabditis elegans*. The primer sequences used were as follows: forward, 5′-GCAGATCGTCATGACCAACC-3′ and reverse, 5′-GTCATGTCGCTTGCCTTAGG-3′. PCR products were TA cloned into PCR2.1 TOPO vector and sequenced to confirm. For the antisense RNA probe, pCR2.1-p43 was linearized with *Bam*HI (NEB) and transcribed with T7 RNA polymerase (Promega, WI, USA). For the sense RNA probe, pCR2.1-p43 was linearized with *Not*I (NEB, MA, USA) and transcribed with SP6 RNA polymerase (Promega, WI, USA). In both cases, digoxigenin (Dig) RNA labeling mix (Sigma-Aldrich, UK) was used to label each probe with a Dig epitope.

Paraffin sections were dewaxed twice for 10 min in fresh xylene and then slowly rehydrated by consecutive washes for 5 min in 100%, 90%, and 70% ethanol. Sections were then washed twice for 5 min in diethyl pyrocarbonate-treated water. FISH on the rehydrated sections was carried out as described^31^. Dig epitopes were detected by tyramide amplification using TSA Plus Cyanine 3 kit (Perkin-Elmer, MA, USA). The slides were visualized using an inverted confocal microscope, Olympus Fluoview FV-100.

### Electron microscopy

The samples were fixed with 4% formaldehyde and 2.5% glutaraldehyde in 0.1 M 4-(2-hydroxyethyl)-1-piperazineethanesulfonic acid (HEPES) buffer (pH 7.2). They were then post fixed with 1% osmium tetroxide, 1.5% potassium ferrocyanide in 0.1 M cacodylate buffer (pH 7.2) for 1 h, and then overnight in 1% uranyl acetate in water. The samples were dehydrated in an ethanol series infiltrated with TAAB 812 resin (TAAB, Berks, UK) and polymerized for 24 h at 60 °C. Sections were cut with Reichert Ultracut ultramicrotome and observed with FEI Tecnai 12 Biotwin microscope (OR, USA) at 100-kV accelerating voltage. Images were taken with Gatan Orius SC1000 CCD camera.

For immunoelectron microscopy, the samples were fixed with 4% formaldehyde and 0.1% glutaraldehyde in 0.1 M HEPES buffer (pH 7.2). After dehydration in an ascending gradient of ethanols, specimens were infiltrated with LR White (London Resin Company, Reading, UK) and polymerized at 40 °C for 48 h. Ultrathin sections were cut with Reichert Ultracut ultramicrotome. For immunolabeling, sections were washed with 0.1 M phosphate buffer, blocked with 1% bovine serum albumin (BSA) in 0.1 M PBS, and incubated with rabbit anti-p43 (Cambridge Research Biochemicals, Billingham, UK) for 1 h. Then, sections were washed with 0.1 M PBS and incubated with anti-rabbit conjugated with colloidal gold particles (SKU# EM:PAG15, BBI Solutions, Tebu-bio, Cambs, UK). They were fixed with 1% glutaraldehyde and after washing with distilled water were stained with 1% uranyl acetate. Samples were observed with FEI Tecnai 12 Biotwin microscope at 100-kV accelerating voltage. Images were taken with Gatan Orius SC1000 CCD camera.

### p43-Native p43 isolation

*Trichuris muris* adult worms were collected from the cecum of mice on day 42 post infection. They were cultured for up to 24 h in RPMI-1640 plus 500 international units/ml (IU) penicillin and 500 μg/ml streptomycin (Invitrogen, Thermo Fisher Scientific, UK). The culture medium containing E/S proteins was pelleted at 720 × *g* for 15 min to remove eggs. E/S in 50-ml Falcon tubes (Corning) was filtered through a 0.22-μm syringe filter (Sartorius) and incubated overnight with end-over-end mixing at 4 °C with 5 mM imidazole and 2 ml of Ni NTA agarose (Qiagen, Manchester, UK). The next day, two washes were performed using 25 mM Tris, 150 mM sodium chloride, pH 7.9, and 20 and 40 mM imidazole. p43 was eluted from the beads in 250 mM imidazole as 10 × 1-ml fractions. The eluate was run on a gel to check for p43 in the fractions. It was then further purified on a size-exclusion column Superdex 75 (GE Healthcare) in 20 mM Tris and 50 mM NaCl, pH 7.9. Zero point five milliliter fractions were eluted and run on 4–12% Bis-Tris gel to confirm the p43-containing fractions. p43 was concentrated using a centrifugal filter unit, 10-kDa MWCO (Millipore), and sterilized using a syringe filter with a 0.22-μm pore size. p43 concentration was determined using a Nanodrop (Labtech International, UK), and protein was aliquoted and stored at −80 °C, until required for experimental use.

### Excretory/secretory products without p43

E/S products that did not bind to nickel beads in the overnight incubation, was termed E/S^−p43^. This was concentrated using a centrifugal filter unit, 10-kDa MWCO (Millipore), and then buffer exchanged into PBS, pH 7.4. Later, it was filter sterilized using a syringe filter with a 0.22-μm pore size, aliquoted, and then stored at −80 °C until use. Removal of p43 was confirmed using SDS-PAGE.

### r43—Recombinant, baculovirus-derived p43 production

The p43 gene minus signal peptide (aa 18–394) was amplified using a DNA template originally from a *T. muris* cDNA library using the following PCR primers: forward, 5′-CTCGTCGGATCCCGGTCACGTAAAATGTCCGGAC-3′ and reverse primer, 5′-CTCGTCGAATTCTCAGTGGTGGTGGTGCTCCTTG-3′. The PCR was carried out using *Pfu* (Strategene, CA, USA) with the following conditions: 94 °C for 1 min, followed by 30 cycles of 94 °C for 15 s, 55 °C for 30 s, and 72 °C for 1.5 min, and then 72 °C for 10 min and 4 °C hold. The PCR was resolved on a 1% agarose gel and excised (Qiagen Gel Extraction Kit), and then digested with *Bam*HI and *Eco*RI enzymes (Fermentas, Thermo Fisher Scientific) and purified using a PCR cleanup column (Qiagen, Manchester, UK). Baculovirus transfer plasmid pAcGP67 (Pharmingen) was also digested with both enzymes and the linearized vector gel purified (Qiagen, Manchester, UK). Ligation was performed using T4 DNA ligase (NEB) and ligation reactions transformed into competent JM109 cells. Sequence confirmation of the construct was carried out using both forward- and reverse-specific vector primers (GATC).

For insect cell protein expression and purification, pAcGP67:*T. muris* p43 (18–394) construct was co-transfected into Sf9 cells with flashBAC ultra DNA (Oxford Expression Technologies). Secreted virus was removed after 4 days and used to amplify 50 ml of Sf9 cells (2 × 10^6^ cells/ml) in a 250-ml sterile Erlenmeyer flask for a further 5 days (120 r.p.m. at 28 °C). Virus was titrated using a conventional plaque assay. Hi5 cells were infected with recombinant *T. muris* p43 virus at a multiplicity of infection (MOI) of 5 and left for 72 h to secrete. Media were collected and cells were removed by centrifugation (720 × *g* for 5 min). Secreted protein was purified from the media as for p43, although no size-exclusion purification step was necessary. Prior to crystallogenesis, selenomethionine (SeMet) labeling of p43 was undertaken. Hi5 cells (Invitrogen) were first adapted to growth in ESF921 media lacking methionine (Expression Systems) by growing for at least six passages: Cells were grown to a density of 2 × 10^6^/ml and infected with recombinant p43 baculovirus. After 16 h, dl-selenomethionine (Generon) at 50 mg/l was added and the culture was left for a further 48 h.

### Crystallogenesis

Recombinant SeMet-derivatized p43 was crystallized by sitting-drop vapor diffusion. The purified protein was concentrated to a final concentration of 8 mg/ml in 20 mM Tris/HCl at pH 7.9. Two-hundred nanoliters of protein was mixed with an equal volume of reservoir solution from five commercially available crystallization screens (JCSG-plus, PACT Premier, Clear Strategy I + II and Morpheus) using a Mosquito (TTP) nanoliter pipetting robot and incubated at 4 **°**C for 16 h.

Following primary crystallogenesis, Matrix seeding^32^ was used to rapidly optimize the available crystals. Initial crystal hits were aspirated along with 50 μl of reservoir solution and added to a Seed Bead apparatus. The seed stock was then generated by vortexing for 90 s before being placed on ice and used immediately without any further dilution. The matrix-seeded sitting drops were set with 180 nl of protein, 20 nl of seed stock, and 200 nl of reservoir solution. These drops were then incubated at 4 °C for a further 16 h. Following incubation, single crystals suitable for X-ray analysis were harvested from (0.2 M lithium sulfate, 0.1 M Tris, pH 8.5, and 40% Peg400 [JCSG D7]) and flash frozen by plunge freezing in liquid nitrogen. Native p43 was also crystallized and optimized in a similar manner to that described above. Single crystals suitable for X-ray analysis were harvested from Morpheus G9 and flash frozen by plunge freezing in liquid nitrogen.

### Data collection and structure determination

Data were collected from single cryo-frozen crystals of SeMet p43 at Diamond Light Source Beamline (i03). A complete dataset was collected at a wavelength of (0.9795 Å) and subsequently scaled and merged using Xia2^33^. The structure was determined using single-wavelength anomalous dispersion in SHELX. An initial model was autobuilt, which clearly described the main secondary structural elements of p43. Data were also collected from a single cryo-frozen crystal of natively expressed p43 at Diamond, Beamline (i03). A complete dataset was collected and subsequently scaled and merged using Xia2. Following initial phasing, the partial autobuilt structure was used as the basis for molecular replacement against the native data. A model of the native p43 structure was subsequently completed and refined using iterative cycles of rebuilding and refinement in COOT^34^ and Phenix.refine^35^. Validation with Molprobity and PDB-REDO was integrated into the iterative rebuilding and refinement process^36^. Complete data collection and refinement statistics are presented in Supplementary Table 2. The crystal structure of p43 contains two monomers in the asymmetric unit; analysis with both size-exclusion chromatography and PISA (http://www.ebi.ac.uk/pdbe/prot_int/pistart.html) indicates that p43 is unlikely to form higher-order assemblies in solution^37^.

### Microscale thermophoresis

p43 (20 µM) was labeled with NHS-647 red dye (NanoTemper, Cambridge), using a 3:1 ratio of dye to protein, and the reaction was left for 30 min at room temperature. Unbound NHS-647 was removed from that bound to p43 using a 5-ml desalting column pre-equilibrated in sample buffer (20 mM HEPES, 150 mM NaCl, and 0.005% Tween-20, pH 7.4). The final concentration of labeled p43 was ~5 µM and this was used as the stock solution for binding experiments. IL-13 (recombinant murine IL-13, PMC0135, Thermo Fisher, USA) was resuspended to a concentration of 40 µM in sample buffer, and a serial dilution from 2500 nM to 40 pM was incubated with 5 nM p43, as per the instructions on the instrument software. Samples were loaded into premium coated capillaries and analyzed with an LED power (which regulates the level of fluorescence) of 5% and an MST power (which creates the temperature gradient for MST to occur) of 40% on a Monolith Pico instrument (NanoTemper). The response amplitude of MST change was ~9.5, and the signal-to-noise ratio was ~19. The sample was run with three different reactions and the results fitted with a K_D_ model (1:1 stoichiometry) within the MO-Analysis software (NanoTemper, Germany).

### Native mass spectrometry

Twenty micromoles p43 in PBS was buffer exchanged 4 times into 250 mM ammonium acetate, pH 7, using Biospin-6 columns (Bio-Rad, USA). Lyophilized recombinant mouse IL-13 was dissolved in 250 mM ammonium acetate, pH 7, and buffer exchanged once in order to remove non-volatile salt adducts. For the binding experiments, p43 and IL-13 were mixed at a ratio of 1.5:1, respectively, and left to incubate at room temperature for 2 h prior to analysis.

All experiments were performed using nano-electrospray ionization in positive ionization mode. Mass spectra and arrival time distribution profiles were recorded on a Synapt G2-Si traveling-wave ion-mobility mass spectrometer (Waters, Manchester, UK). Typical source parameters utilized were as follows: capillary voltage, ~0.9–1.2 kV; sample cone, ~20 V; source temp, 60 °C; Trap gas, 4 ml/min. Stepwave voltages were also optimized so as to promote ion transmission and sample cleanup while minimizing structural perturbations. Ion charge states corresponding to specific *m*/*z* values were mass selected within the quadrupole prior to CID experiments. CID was carried out with argon as the collision gas within the trap cell region. Data were acquired and processed with MassLynx V4.1 software (Waters, Manchester, UK). Mass spectra were deconvoluted using the UniDec software^38^. Error in the calculated experimental masses of p43 and the p43-IL-13 complex were obtained from the full-width half-maxima for the center of mass of both species.

### Surface plasmon resonance

SPR was used to investigate the binding of p43 to HS. HS was biotinylated at the reducing end by reductive amination^39^. Briefly, HS (low molecular weight fraction-1 from porcine mucosa, Iduron, UK) was dissolved in 2 M ammonium chloride (NH_4_Cl), in a volume of 100 μl. Two milligrams of sodium borocyanohydride (NaCNBH_3_) was added, and the mixture was heated at 70 °C for 2 days. Following dialysis into PBS, 10 μl of 3 mg/ml sulfosuccinimidyl-6-(biotinamido) hexanoate (Sulfo-NHS-LC-Biotin, Thermo) was added and incubated overnight at 4 °C. Non-reacted biotin was then removed by further dialysis into 0.1 M sodium acetate, pH 5.5.

Streptavidin sensors (SA-chips) were used in a Biacore T200 instrument (GE Healthcare) with a running buffer of 10 mM HEPES, 150 mM NaCl, and 0.005% Tween-20 both with and without zinc. For kinetic analysis, the surface was loaded with ~50 U of Biotin-HS in running buffer with 50 µM zinc chloride. p43 was injected at concentrations of 0, 2, 5, 10, 15, 20, and 25 nM with an 80-s injection and a 600-s dissociation before regeneration with 10 mM ethylenediaminetetraacetic acid (EDTA). Analyte sensograms were analyzed in BIAevaluation version 3.1 with a 1:1 Langmuir model fitted with a global R-Max.

Investigation into the dependence of ions on p43 binding to HS was conducted on a Biotin-HS surface of ~100 U of HS. In these experiments, the running buffer did not contain zinc. p43 was injected at a concentration of 100 nM in solutions containing running buffer plus 20 µM of cobalt, copper, iron, nickel, zinc, calcium magnesium, and manganese. In each case, the sample was injected for 60 s, and immediately the HS surface was washed with 10 mM EDTA prior to injection of the next sample. Total binding was selected as the response level at 60 s following injection.

IL-13 competition was performed using a surface coverage of ~200U of biotinylated HS in running buffer with 50 µM zinc chloride, ZnCl_2_ p43 was injected at a concentration of 100 nM for 60 s before injection of IL-13 at a concentration of 1 µM diluted in running buffer plus 50 µM ZnCl_2_. Inhibition of p43 binding to HS was performed on the same HS surface by injection of p43 as the analyte with the addition of increasing concentrations of IL-13 to p43 prior to injection. Samples were incubated for 10min prior to injection.

### Peritoneal cell isolation

Female, C57BL/6 mice were injected intraperitoneally (i.p.) with 4% thioglycolate (Becton Dickinson). Three days later, mice were killed under CO_2_ and 5–7 ml of ice-cold RPMI-1640, 5% fetal calf serum (FCS), was injected i.p. After massaging, the injected medium was withdrawn and the cells were collected in a 15-ml tube (Corning). The cells were pelleted and washed once with the medium before counting using a CASY automated cell counter. A total of 1 × 10^6^ cells/ml were plated in a 24 sterile tissue culture plate. After 3 h, the medium was replaced by either fresh medium, medium containing 20 ng/ml of recombinant mouse (rm)IL-13, 20 ng/ml rmIL-13, and 20 μg/ml of p43 or 20 μg/ml p43 alone. In a repeat experiment, 20 ng/ml rmIL-4 and IL-4 plus p43 were used. All conditions contained 100 μg/ml HS and 100 mM zinc (ZnCl_2_). The plates were left at 37 °C in 5% CO_2_. Forty-eight hours later, the cells and medium were taken, centrifuged to pellet the cells, and the supernatant was assayed for RELM-α by ELISA.

### RELM-α ELISA

ELISA plates (Greiner, GmbH, Austria) were coated at 1 μg/ml with rabbit anti-mouse RELM-α (Peprotech, UK) and incubated overnight at 4 °C. The next day, the plates were washed 5× with PBS + 0.05% Tween-20, and a blocking step was carried out using PBS + 10% FCS for 1 h at room temperature. Supernatants from peritoneal macrophages were diluted and added to the plates for 1 h. After washing again, the plates were incubated with 1/200 biotinylated rabbit anti-mouse RELM-α (Peprotech, UK) for 1 h at room temperature. The plates were subsequently washed and developed with 3,3′,5′,5-tetramethylbenzidine. The reaction was stopped with 0.18 M H_2_SO_4_, and the plates were read on a VersaMax ELISA reader (Molecular Devices, CA, USA), and the data were analyzed using the SoftMax Pro 6.4.2 software.

### Intranasal administration of IL-13 ± p43, zinc, and HS

PBS, 5 ng of recombinant murine IL-13, and 45 μg of r43 were given intranasally (i.n.), singly, or in combination together with or without Zn and HS. All preparations were made up and stored at 4 °C overnight before administration. Fifty microliters were administered to each mouse under anesthesia 24 h prior to killing.

### Isolation of immune cells from the lung

Following killing, bronchoalveolar cells were obtained by washing of the cavity or lungs with PBS containing 2% FCS (Sigma) and 2 mM EDTA (Thermo Fisher). Lungs were processed as previously described^20^, and incubated at 37 °C for 40 min with 0.8U/ml Liberase TL (Roche Diagnostics) and 80 U/ml DNase I type VI in HBSS (both Sigma). The digestion was stopped with PBS containing 2% FCS (Sigma) and 2 mM EDTA (Thermo Fisher), and the resulting suspension was then passed through a 70-μm cell strainer. Erythrocytes were lysed using red blood cell lysis buffer (Sigma), and cells were counted and processed for flow cytometry.

### Flow cytometry

Equal numbers of cells were stained for each sample, washed with ice-cold PBS, and stained with Zombie Ultraviolet dye (BioLegend) for 10 min at room temperature. All samples were then blocked with 5 μg/ml αCD16/CD32 (2.4G2; BioLegend) in FACS buffer (PBS containing 2% FCS and 2 mM EDTA) before staining for specified surface markers at 4 °C for 25 min. For detection of intracellular molecules, following surface staining, cells were fixed with 1% paraformaldehyde in PBS for 10 min at room temperature, permeabilized with the Transcription Factor Staining Kit (eBioscience), and then stained with the anti-RELM-α FITC antibody (Peprotech, UK). Samples were acquired using a 5 laser Fortessa with BD FACSDiva software and analyzed with the FlowJo software (v9, Tree Star).

### Infection regimes

For high-dose infections, C57BL/6 mice were infected at day 0 with 200 *T. muris* eggs in a volume of 0.2 ml by oral gavage. For low-dose infections, 20 *T. muris* eggs were used. Parasite burden in the cecum was assessed at day 21 p.i. and day 35 p.i. At day 21 p.i., MLNCs were removed, a cell suspension was made, and 200 μl of 5 × 10^6^ cells/ml were restimulated in vitro with 50 μg/ml of whole E/S, p43, or E/S^−p43^. Cell supernatants were taken at 48 h and pelleted to remove cells. The supernatants were analyzed for cytokine content. At day 35 p.i., mice were exsanguinated by cardiac puncture. The blood was allowed to clot and serum was obtained by centrifuging the blood. Serum was then used for parasite-specific antibody ELISA analysis. Long-term infections were generated by infecting mice with a low-dose infection and terminating the experiment at day 55 p.i. Trickle infections involved a low-dose infection being administered at day 0, 2, 4, 7, 9, and 11 p.i. and terminating the experiment at day 35 p.i.

### Antibody ELISA

Ninety six-well ELISA plates (Greiner GmbH, Austria) were coated with 5 μg/ml whole E/S, purified p43, and E/S^−p43^, all at 5 μg/ml in sodium carbonate bicarbonate buffer, pH 9.6, and incubated at 4 °C overnight. The next day, plates were washed with PBS + 0.05% Tween-20 five times using an automated ELISA plate washer (Molecular Devices). Plates were incubated with 100 μl of 3% BSA (Sigma) in PBS with Tween-20 for 1 h and washed as previously described. Fifty microliters of serum from C57BL/6 mice given either 200 (high-dose) or 20 (low-dose) *T. muris* eggs and collected on either day 35 (high dose and post trickle) or day 55 (low dose), was diluted 1/80 in PBS + 0.05% Tween-20, and plated onto all three antigens. After a 90-min incubation at room temperature, plates were washed as previously described, and all plates were incubated with goat anti-mouse immunoglobulin (Ig, G, M, and A) conjugated to alkaline phosphatase (A0162, Sigma-Aldrich, UK) at 1/4000 for 60 min. After washing for a final time, plates were developed with 100 μl of *p*-nitrophenyl phosphate. The plates were read on a VersaMax ELISA reader and analyzed using SoftMax Pro 6.4.2 software.

### In vitro re-stimulation

MLNs were removed from infected C57BL/6 mice at day 21 p.i., and a cell suspension was prepared in RPMI-1640, 10% FCS, 100IU/ml penicillin, 100 μg/ml streptomycin, and 2 mM l-glutamine (all Sigma-Aldrich, UK). The cells were resuspended at 5 × 10^6^ cells/ml and plated in 96-well tissue culture plates with 50 μg/ml of E/S, p43, or E/S^−p43^. After 36 h, cell supernatants were centrifuged at 19,000 × *g* to pellet cells and supernatants were stored at −20 °C until analysis.

### Cytokine analysis

Cytokine levels in MLNC supernatants were analyzed by Cytokine Bead Array Flex Sets™ for IL-13 (558349) and IFN-γ (558296, both from Becton Dickinson). Capture beads conjugated to IL-13 and IFN-γ were incubated with supernatants and a dilution range of recombinant murine standards and allowed to mix at room temperature on a rocker for 1 h. Phycoerythrin-conjugated antibodies were added directly onto the supernatants or standards and their capture beads and incubated again for 1 h on a rocker. The beads were then pelleted by centrifugation, washed, and resuspended with BD wash buffer. The assay was then read on a MACSquant flow cytometer (Miltenyi) and analyzed using the BD FACSarray software.

### Vaccination protocol

Antigens, p43 and E/S^−p43^, were prepared for vaccination by mixing 1:1 with alum (Imject, Thermo Fisher). The antigen was added dropwise to alum and mixed for 30 min. C57BL/6 mice were administered subcutaneously at 50μg per vaccination (100 µl of vaccine) on day −28 and boosted on day −14. They were then infected with 20 *T. muris* eggs on day 0. Mice were culled, blood was taken, and worm burdens were assessed on day 35 p.i.

### Statistical information

All data are shown as the mean ± SEM or SD when stated. For groups of three or more, a one-way analysis of variance was performed. GraphPad PRISM v.7.0 was used for statistical analysis and a value of *p* < 0.05 was considered significant. For qPCR data shown in Supplementary Fig. 1c, Kruskal–Wallis test was performed.

### Reporting summary

Further information on research design is available in the Nature Research Reporting Summary linked to this article.

## Supplementary information

Supplementary Information

Reporting Summary

Source Data

## Data Availability

All the data needed to make the conclusions from this study are present in the main paper or supplementary data. Any additional raw data can be obtained by emailing the corresponding authors. “Coordinates and structure factors have been deposited in the Protein Data Bank under accession code 6QIX.”
