# Supplementary material for: The major secreted protein of the whipworm parasite tethers to matrix and inhibits interleukin-13 function
Source: Nat Commun. 2019 May 28;10:2344. doi: 10.1038/s41467-019-09996-z (PMC6538607; doi:10.1038/s41467-019-09996-z)
Supplement: Supplementary file 1 — Supplementary Information [file 41467_2019_9996_MOESM1_ESM.pdf]

Supplementary Data: The major secreted protein of the whipworm parasite tethers to matrix and inhibits interleukin-13 function

Authors: Allison J Bancroft[1,2,3,4*], Colin W Levy[5], Thomas A Jowitt[2,4], Kelly S Hayes[1,2,3,4], Seona Thompson[1,2,3,4], Edward A Mckenzie[5], Matthew D Ball[5], Eamon Dubaissi[1,2,3,4], Aidan P France[5], Bruno Bellina[5], Catherine Sharpe[1,2,3,4], Aleksandr Mironov[4], Sheila L Brown[1,3,4,6], Peter C Cook[1,3,4,6], Andrew MacDonald[1,3,4,],[6] David J Thornton[1,2,3,4], and Richard K Grencis[1,2,3,4,*]

*Corresponding Authors: Allison J Bancroft (allison.j.bancroft@manchester.ac.uk) and Richard K Grencis (Richard.Grencis@manchester.ac.uk)

Supplementary Figure 1

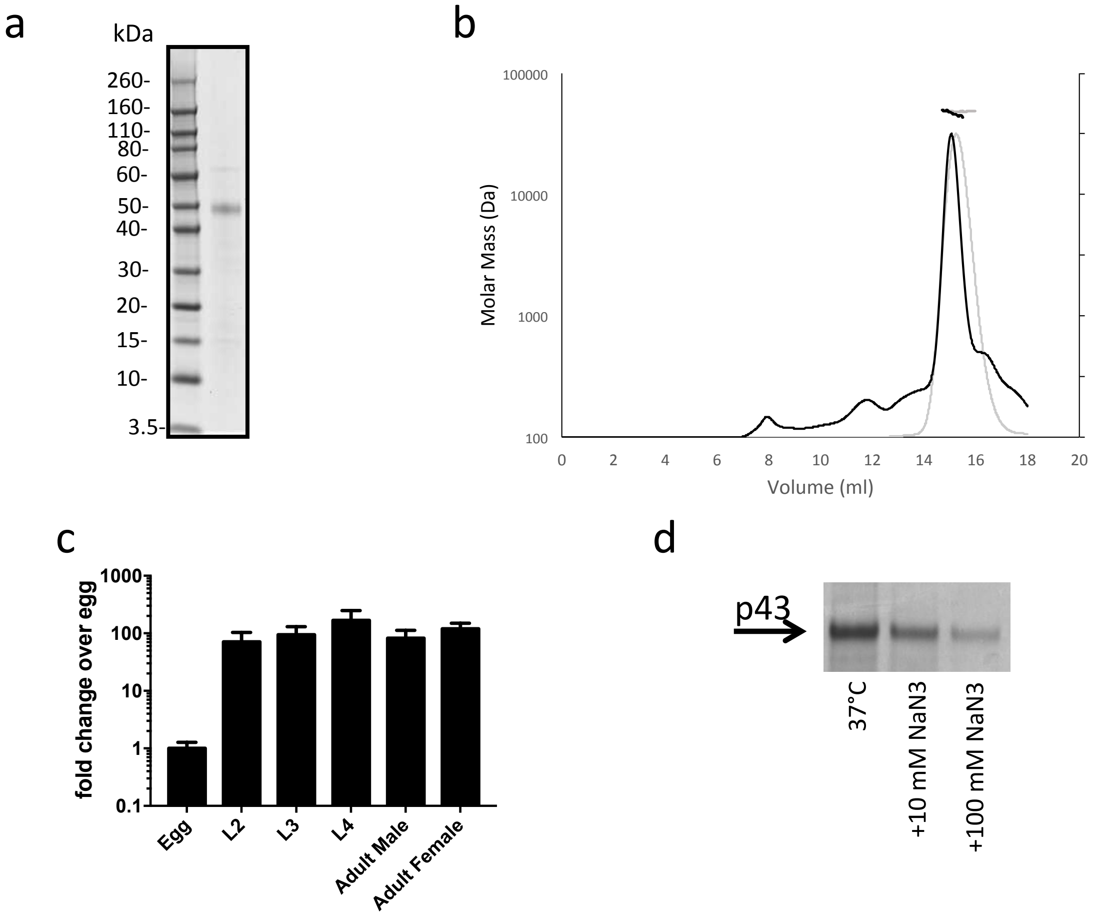

Supplementary Figure 1-The major E/S protein of *T. muris* is a single dominant protein of approximately 43 kDa. The p43 gene along with myosin is expressed in all life cycle stages and secretion is reduced by sodium azide.

a SDS PAGE of E/S from cultured adult *T. muri*s worms. **b** Multi angle light scattering (MALS) of native whole E/S from adult *T. muris* (black trace) and native purified p43 (grey trace) revealing a major protein of 46.9 kDa and 48.54 kDa respectively. The graph shows the refractive index and is a measure of all soluble material eluted from the column. The lines above the peak are the mass across the peak. **c** qPCR of p43 in all life cycle stages normalized to 18s RNA and referenced to egg/L1. Error bars are SEM of 3 biological replicates and the data were analyzed by Kruskal-Wallis with Dunns multiple comparison test. **d** 10 adult female and 15 male *T. muris* worms were cultured and10 µl of culture medium was run on a 4-12% SDS PAGE. The MALS shown in **b** and the sodium azide experiment in **d** are representative of at least 3 independent experiments. qPCR shows 3 biological and 3 technical replicates, error bars are standard error. Uncropped gels from this figure a & d are shown in Supplementary Figure 11.

Supplementary Figure 2a

ATGGTAGCAATGCTTGTGCTCTTCTTTCCGCTACTGCTGACGGTTGGCCTGTCCAC
CGCTGGTCACGTAAAATGTCCGGACTTCGGCGACTGGAAACCATGGACCGACTG
CCTTTGGTATCCGCCGCAACACATGTACTCGAAACTGTCGCACGCCTGCGGCATG
CACGCCCACCGCAACCTAACCGGCGTCATGGATCTGCCGCACGGACACAAGACA
CCACCGCCGTGCGGTCATTGCAGTTTTAAATTCCGATGCCGCCGAAGGCCCAACA
CTGAGGGCTGCTACCCGCTCGACGGCGAAGTGGAGGTGTGCCACGATCACAGCG
ACATCTGCACGCTGCCCAAGTTGCCTCACCTGGGCTGCGGCTACGCTTTCATTAA
CGAAAAATTGAAGCAATGCTTCACTCGACCCGACACGCCCTCGTACGTACGCCTC
GGATACCGAAAGATGTTCGAAAGCATCCCCAAAAAGCACTGTATCGAGAAAGAT
GGAATGTGCAAGTGTTGTTGCGGTGACTACGAACCGAACGAGTCGGGCACCGAA
TGCATCAAACCGCCGGCGCACGACTGTCCCGCCTATGGACCACCGAGCGAATGG
AGCGAGTGCCTGTGGTTCCCGTTGAAGAACATTGTCAGCCACGTCTACGACCATT
GTCACGTTCACAAGGAACCCGACGGCTACGAACCGCACAGCGTTGCCCCGGCCA
ACGTGCACATCCCGGAGAAGTGCGGCTTCTGCAGCTTCCGCGTCAAGTGCATGA
AGCGAGACAAGAAGGACGGATGCTTCCCCCTGAAATTGGGCAAGAAGAGTTGCG
GCAAGGACGACTGCCCAACCTGCGGTGACATTTGCACGCTGGACAAGATCAACG
GATCGTGCGCTTTCCCGCGCGTCATGAAGGAGAAAATCTGGGACGACTTCACCG
CCACCAGCAAGGAGAAGCATATGCCTCATTGGAAGCGCGACGGATACGCCAAAA
TGCTAATGCAACTTCCCTACAGCAATTGCAAAGAGGTCGGCGACAAGTGCAAAT
GCTGCTGCCATCCGTACGAGCCGAACAAGGACGGCACCGCCTGCGTTGTCAAGG
AATACTGCAAGCGAGTGCACGAGCTGCACCACCACGATCACCACGGCCACGGA
GAGGAGCACCACAAGAGCAGCAGCAGCGAAAGCAAGGAGCACCACCACCACTG
A

b

MVAMLVLFFPLLLTVGLSTAGHVKCPDFGDWKPWTDCLWYPPQHMYSKLSHACG
MHAHRNLTGVMDLPHGHKTPPPCGHCSFKFRCRRRPNTEGCYPLDGEVEVCHDHSD
ICTLPKLPHLGCGYAFINEKLKQCFTRPDTPSYVRLGYRKMFESIPKKHCIEKDGMCK
CCCGDYEPNESGTECIKPPAHDCPAYGPPSEWSECLWFPLKNIVSHVYDHCHVHKEP
DGYEPHSVAPANVHIPEKCGFCSFRVKCMKRDKKDGCFPLKLGKKSCGKDDCPTCG
DICTLDKINGSCAFPRVMKEKIWDDFTATSKEKHMPHWKRDGYAKMLMQLPYSNC
KEVGDKCKCCCHPYEPNKDGTACVVKEYCKRVHELHHHDHHGHGEEHHKSSSSES
KEHHHH

Supplementary Figure 2-Nucleotide and amino acid sequence of TMUE_ 3000012139. **a** The nucleotide sequence of p43. **b** Amino acid sequence of *T. muris* p43 that shows a poly cysteine, histidine tailed protein.

Supplementary Figure 3

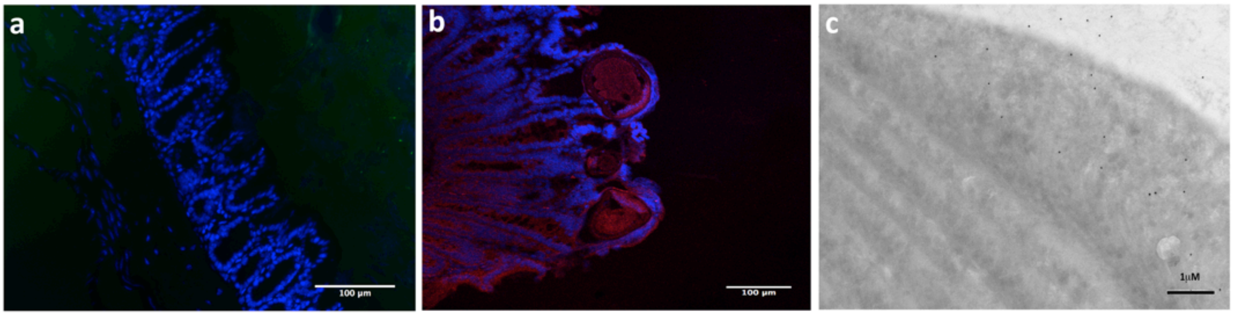

Supplementary Figure 3-Anti-p43 antibody and probes are highly specific. **a** Control immuno staining from Figure 1b-d where pre-immunization serum was used as the primary antibody. **b** Control FISH staining from Figure 1e using the sense probe. **c** Control immuno EM from Figure 1 **f & g** where secondary antibody only was used.

Supplementary Figure 4

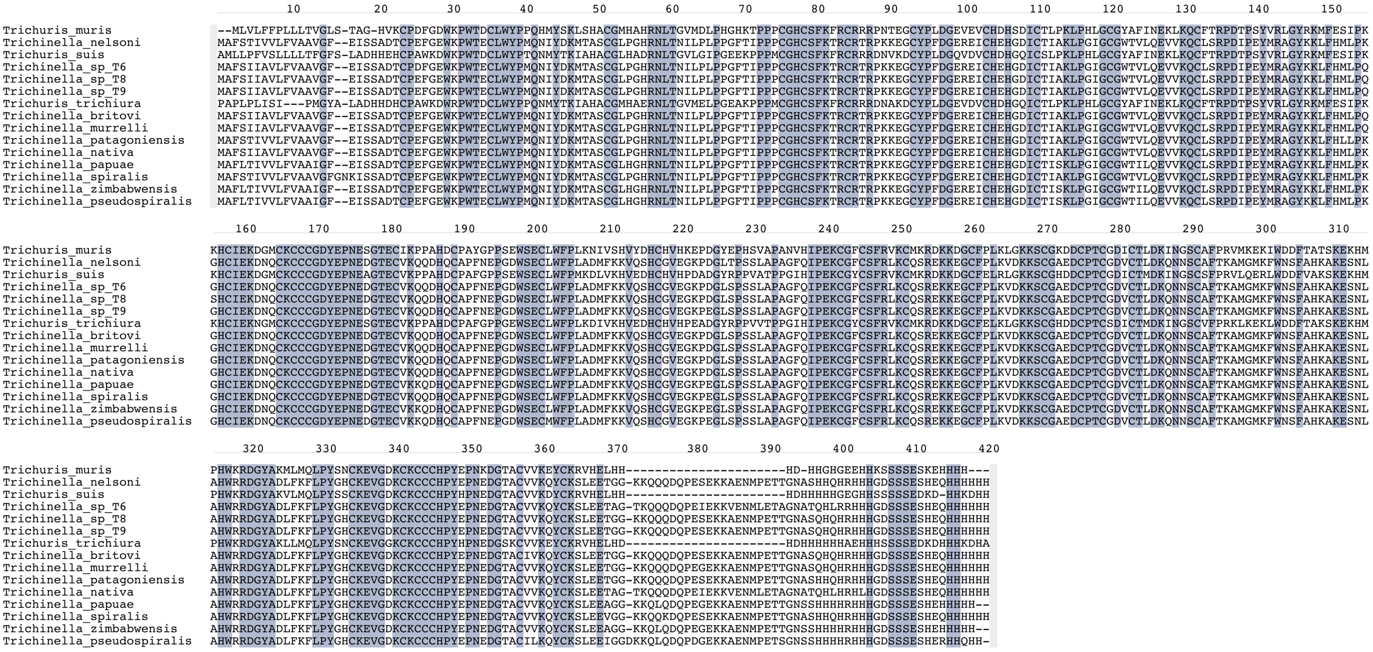

Supplementary Figure 4-TMUE_3000012139 nucleotide sequence is specific to *Trichuris* species and the closely related *Trichinella* species.
The p43 gene is restricted to Clade I nematodes[1] predominantly *Trichuris spp* and the closely related *Trichinella spp*. The grey shaded areas show where there is 100% conservation across the alignment and show a high degree of sequence conservation is observed across the available species. The numbering above the sequence is that of the *T. muris* sequence. The alignment matrix blosum62[2] was implemented as in ICM-Pro. T6, T8 and T9 refer to isolates from grizzly bear, lion and raccoon dog as detailed in [3]

Supplementary Figure 5

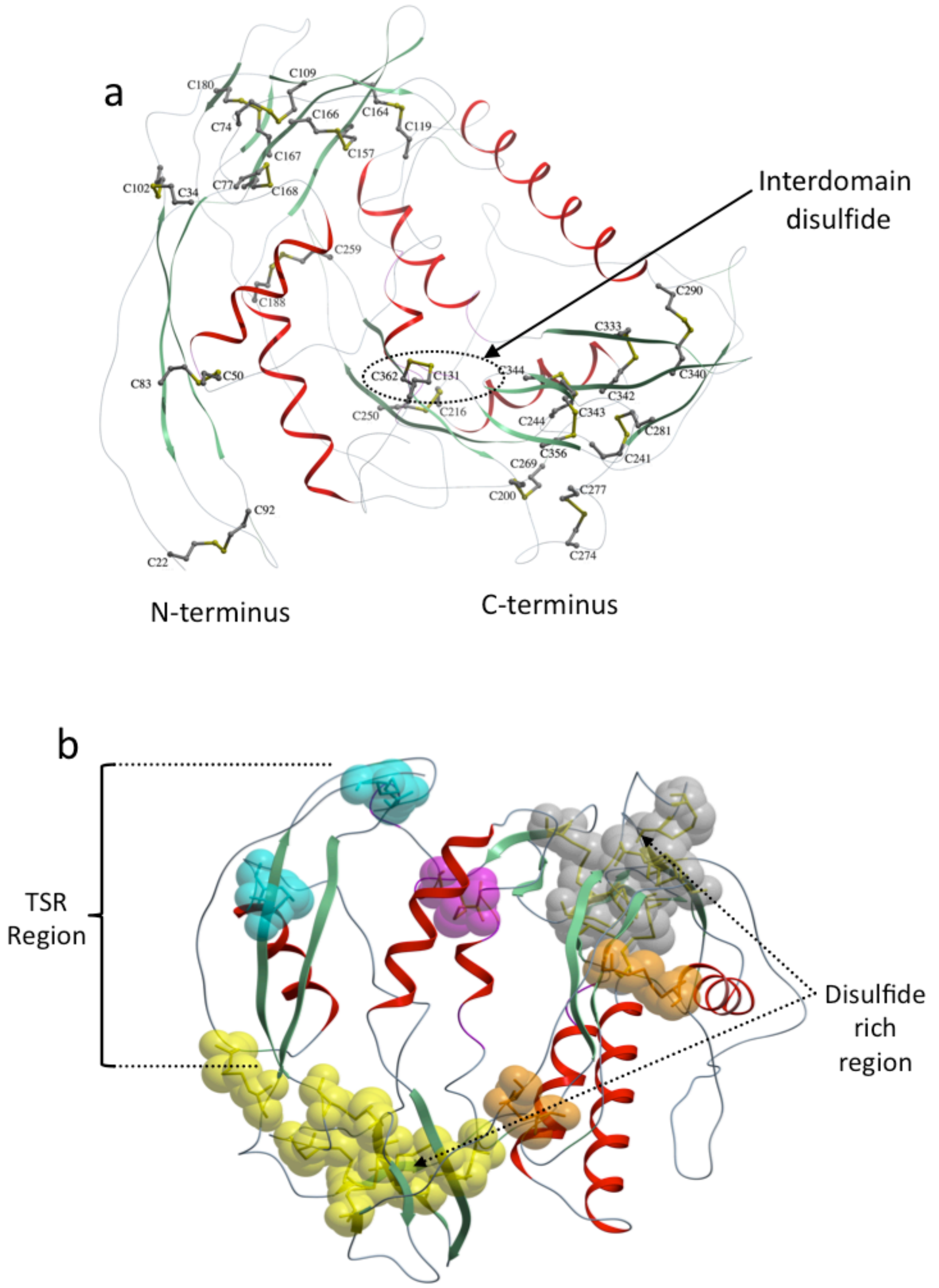

Supplementary Figure 5-The crystal structure of p43 shows interdomain disulfide bonds and a TSR-1 domain.
a A ribbon structure of p43 revealing an interdomain disulfide bond. **b** Position of the functional TSR-1 domain and disulfide rich regions.

Supplementary Figure 6

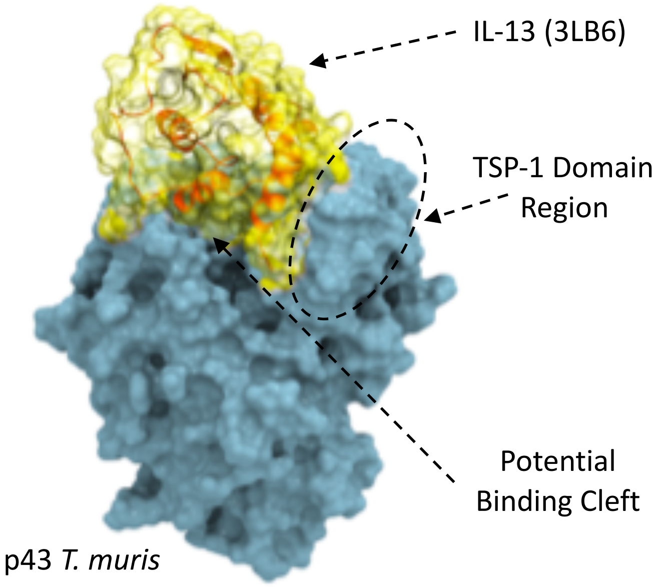

Supplementary Figure 6- Fast Fourier Transform (FFT) Protein-Protein docking simulation between *T. muris* p43 and IL-13. The simulation was performed by firstly extracting the coordinates of IL-13 from the deposited crystal structure of IL-13 in complex with IL-13Ralpha2 (3LB6). p43 was then selected as the receptor whilst IL-13 was defined as the ligand before running a global docking simulation allowing for all possible interactions between the two proteins to be explored. The docked IL13 is shown in both ribbon representation (red) and a semi-transparent surface representation (yellow) occupying a deep cleft in the surface of p43 with close contact to the TSP-1 domain region. p43 is shown in surface representation (blue) with contact patches to IL-13 coloured grey. The docking procedure resulted in several alternative poses for potential IL-13 binding and experimental efforts to validate the docking results presented here are ongoing. The docking simulation was performed using the FFT procedure as implemented in ICM-Pro (http://www.molsoft.com/icm_pro.html).

Supplementary Figure 7

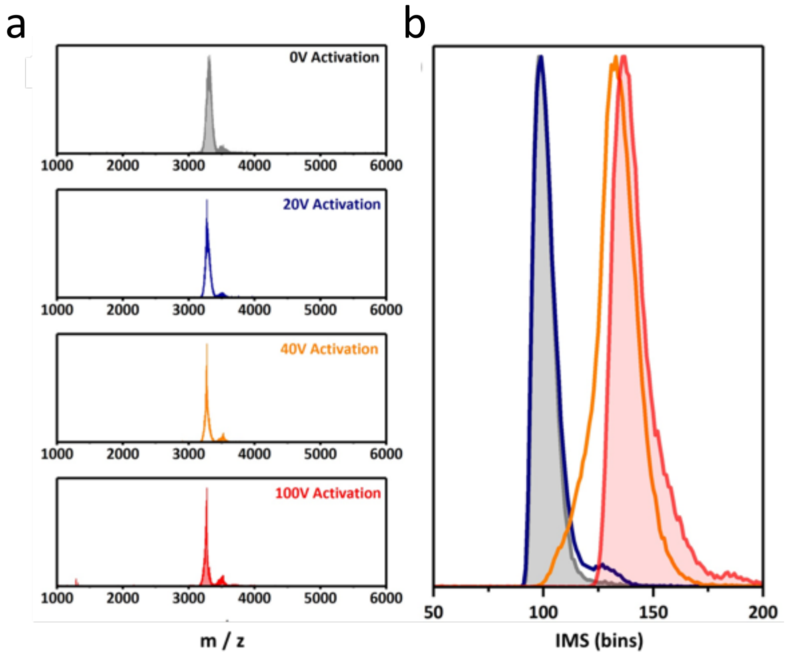

Supplementary 7-Mass spectra of the mass selected p43 14+ ion subjected to different trap collision energies.

a p43 is not particularly prone to fragmentation as there are barely detectable fragments post activation even up to a collision energy of 100 V. **b** Corresponding ion mobility, arrival time distribution (ATD) profiles for the collisional activation of p43 (14+ ion) at 0V (grey), 20V (blue), 40V (orange) and 100V (red). These experiments show that p43 is fairly flexible *in vacuo* and can undergo at least one large conformational change upon activation.

Supplementary Figure 8

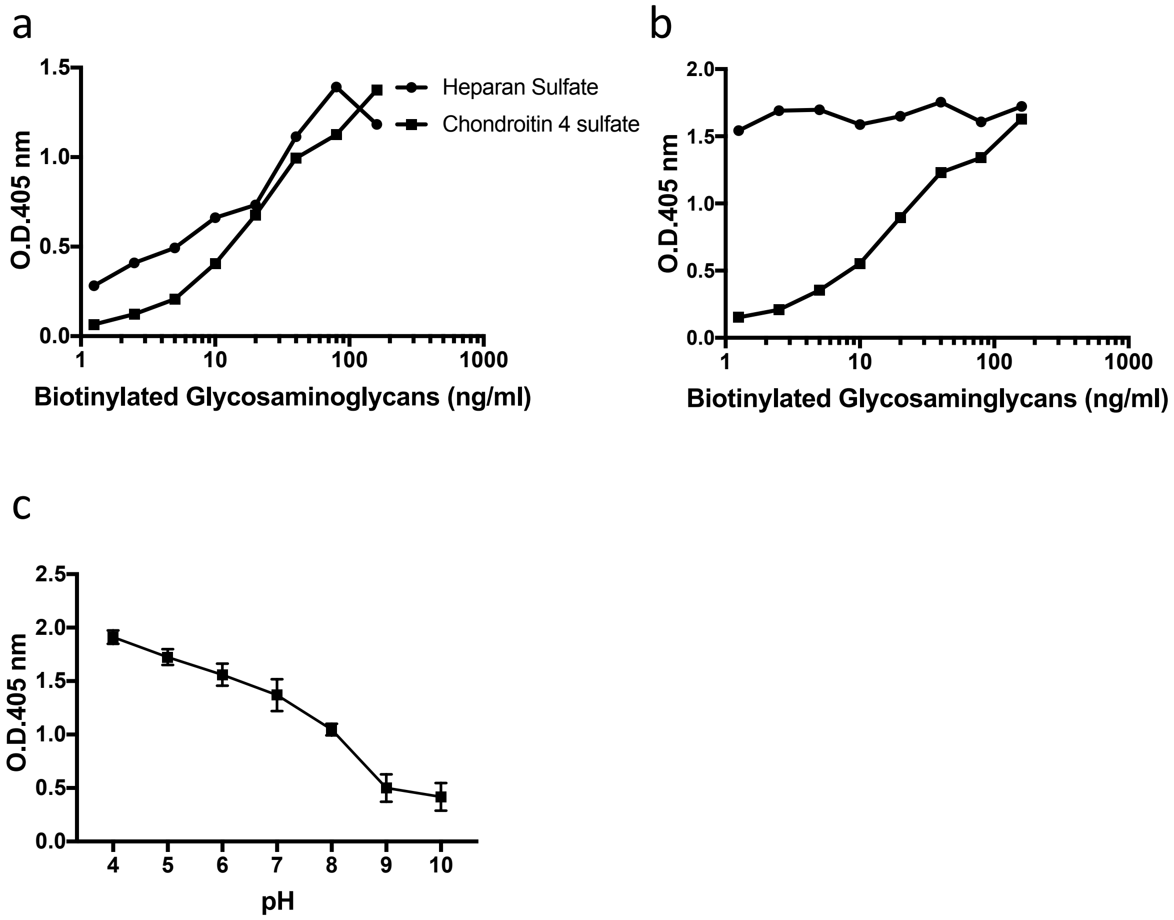

Supplementary Figure 8-p43 and r43 bind to heparan sulfate (HS) by ELISA over a wide pH range.
a & b. Both native p43, **a** and recombinant r43, b bind well to biotinylated glycosaminoglycans, heparan sulfate (HS) and chondroitin 4 sulfate. **c** r43 binds to biotinylated HS over a wide pH range between pH4-7 dropping gradually between pH7-10. These data are representative of at least 3 independent experiments.

Supplementary Figure 9

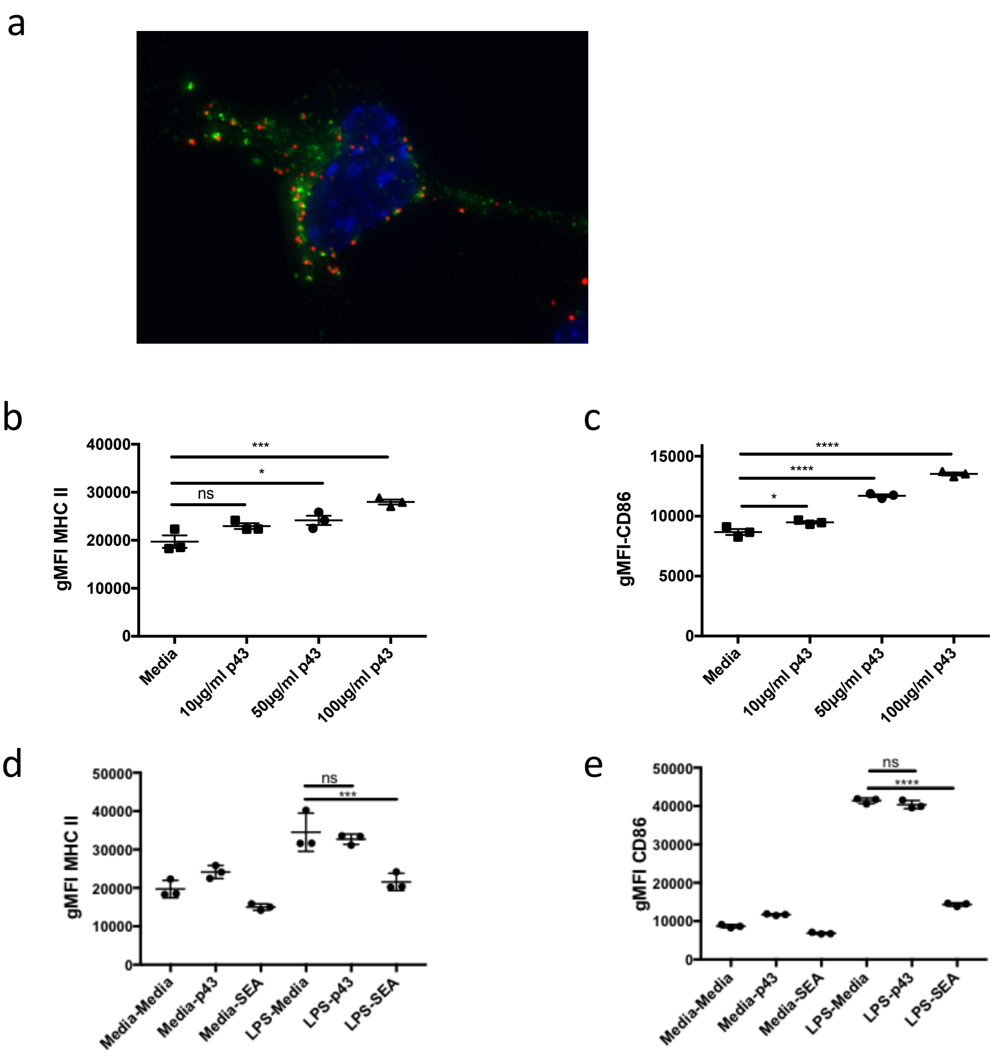

Supplementary Figure 9-p43 can enter dendritic cells and co localize with MHC II. It can activate both MHC II and CD86 in a dose dependent manner.

a Bone marrow derived dendritic cells (BMDDC) incubated with Cy3 labeled p43 (red) and stained with H2-M Alexa Fluor 488 (green). **b & c** BMDDC cultured with different doses of p43 and stained for flow cytometry. CD11C+ cells were stained for **b** MHC II and **c** CD86. **d & e** BMDDC cultured with media and lipopolysaccharide (LPS) and stimulated with p43 or *Schistosoma mansoni* egg antigen (SEA), **d** MHC II and **e** CD86. A one way ANOVA was used to analyse the data, **b** F=14.23, DF=8, Media vs 50µg/mlp43* p 0.0347; media vs 100µg/ml ***p 0.0009; **c** F=192.9, DF=8, media vs 10µg/ml *p 0.0285, media vs 50µg/ml & media vs 100 µg/ml****p<0.0001; ns=not significant; **d** F=25.75, DF=12, LPS-Media vs LPS-p43=not significant(ns)., LPS-Media vs LPS-SEA ***p 0.0006; e, F=2131, DF=12, LPS-Media vs LPS-p43 ns, LPS-Media vs LPS-SEA ****p<0.0001.

Supplementary Table 1

	Identified Proteins (10/497)	MW	Cecal Mucus	Colonic Mucus
1	poly cysteine and histidine tailed protein (p43)	43 kDa	9	2
2	retrovirus_Pol_polyprotein	151 kDa	2	0
3	Gag_Pol_polyprotein	179 kDa	0	5
4	DNA_polymerase_subunit_gamma	136 kDa	2	0
5	DNA_polymerase_zeta_catalytic_subunit	173 kDa	3	0
6	Gag_Pol_polyprotein	148 kDa	0	0
7	copia_type_pol_polyprotein	18 kDa	2	0
8	mediator_of_RNA_polymerase_II	164 kDa	0	2
9	polymerase_(RNA)_II_(DNA_directed)_polypeptide	134 kDa	0	2
10	gag_pol_polyprotein	123 kDa	0	3

Supplementary Table 1-List of the top 10 proteins identified in infected intestinal mucus samples during chronic *T. muris* infection. The list is compiled of the top 10 proteins identified from two independent experiments by tandem mass spectrometry analysis of pooled cecal and colonic mucus samples (n=5 per group) from chronically infected C57BL/6 mice. The numbers in the cecal mucus and colonic mucus columns represents the number of unique peptides identified. The mucus extraction was carried out by PBS and 2M urea incubations (cecum) or flushes (colon), and both flushes were analyzed separately by tandem mass spectrometry analysis before combining (as shown in the table). The top 10 proteins identified by tandem mass spectrometry is based on the number of peptides resolved from the experiment and are displayed in the table. Tandem mass spectrometry data were searched against an in house *T. muris* database, using the Mascot search engine. Scaffold proteome software was used to validate data, using the parameters; 80% protein threshold, 50% peptide threshold and a minimum of 2 unique peptides were required to identify proteins. MW= molecular weight.

Supplementary Figure 10

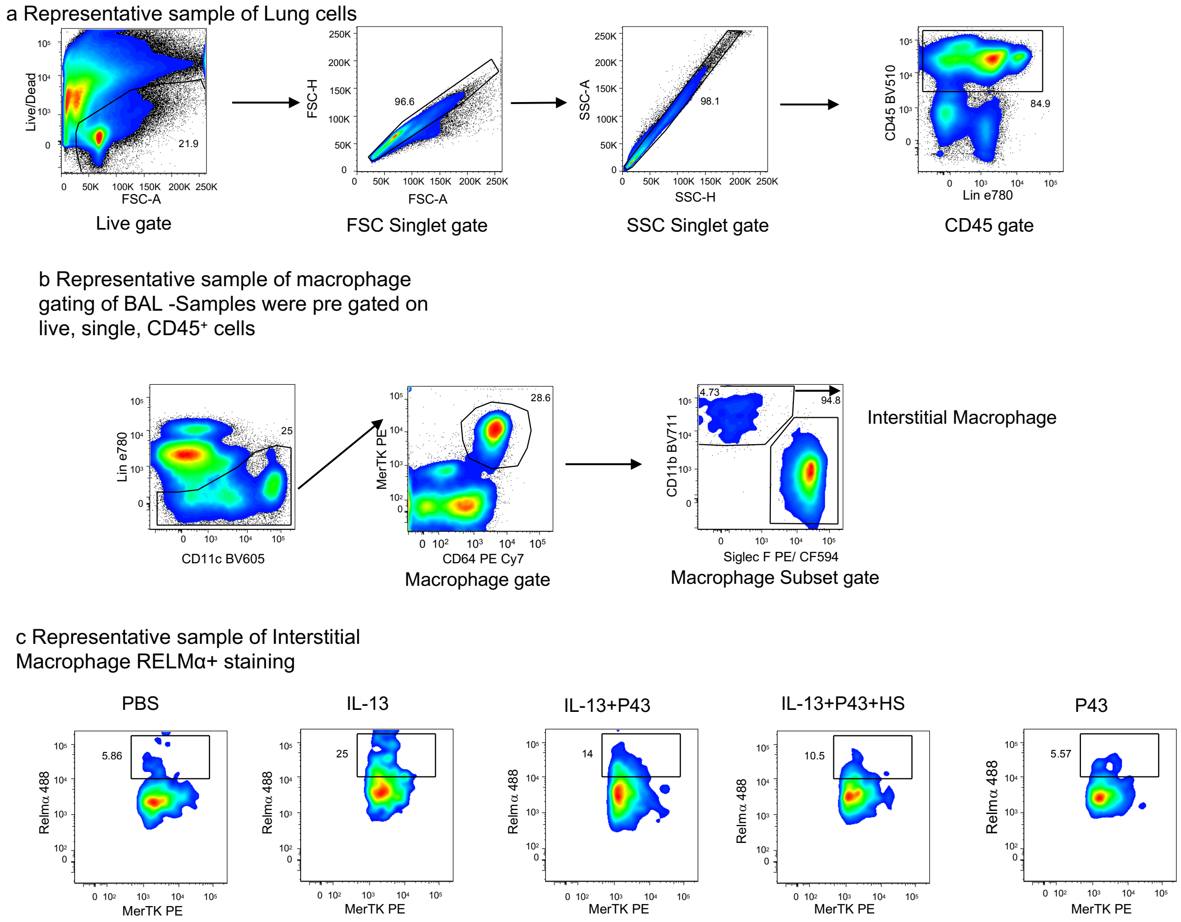

Supplementary Figure 10-Gating Strategy for Interstitial Relm α+ macrophages from Figure 3h. a To identify lung macrophages, flow cytometry analysis of single cell suspensions from digested lung tissue were analysed to remove dead, doublets and CD45- events, and defined as lineage (Lin; CD3,CD19, NK1.1, Ly6G, Ter119)-CD64+MerTK+. Interstitial macrophages were identified as Lin-CD64+MerTK+SiglecF-CD11b+. **c** Examples of Relmα staining of interstitial macrophages from treated mice are shown.

Supplementary Figure 11

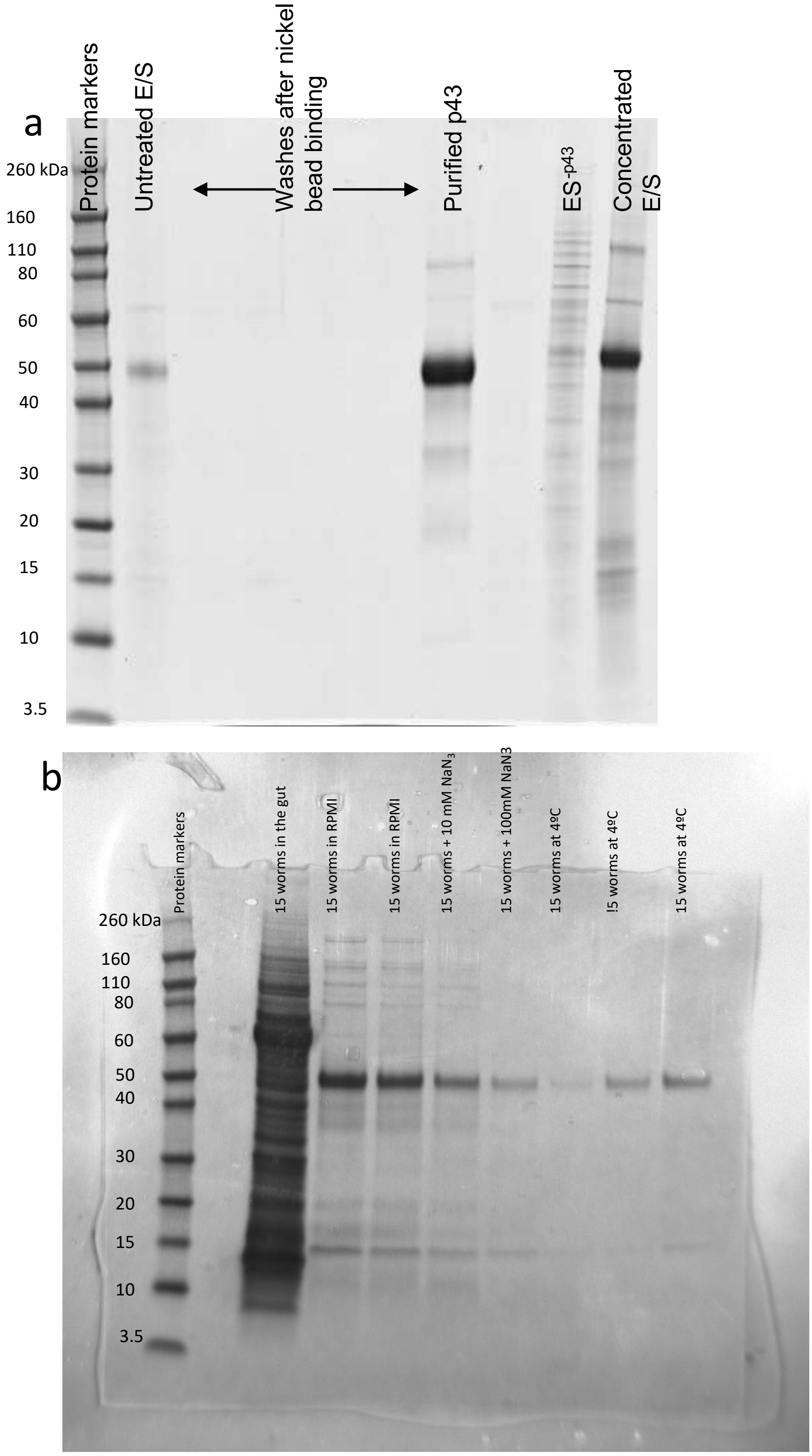

Supplementary Figure 11-SDS PAGE from Supplementary Figure 1a and 1d. SDS PAGE of uncropped SDS PAGE from Supplementary Figure 1 showing **a** that p43 is the single most abundant protein in T. muris E/S and **b** that secretion of p43 is inhibited by increasing concentrations of sodium azide.

Supplementary Figure 12

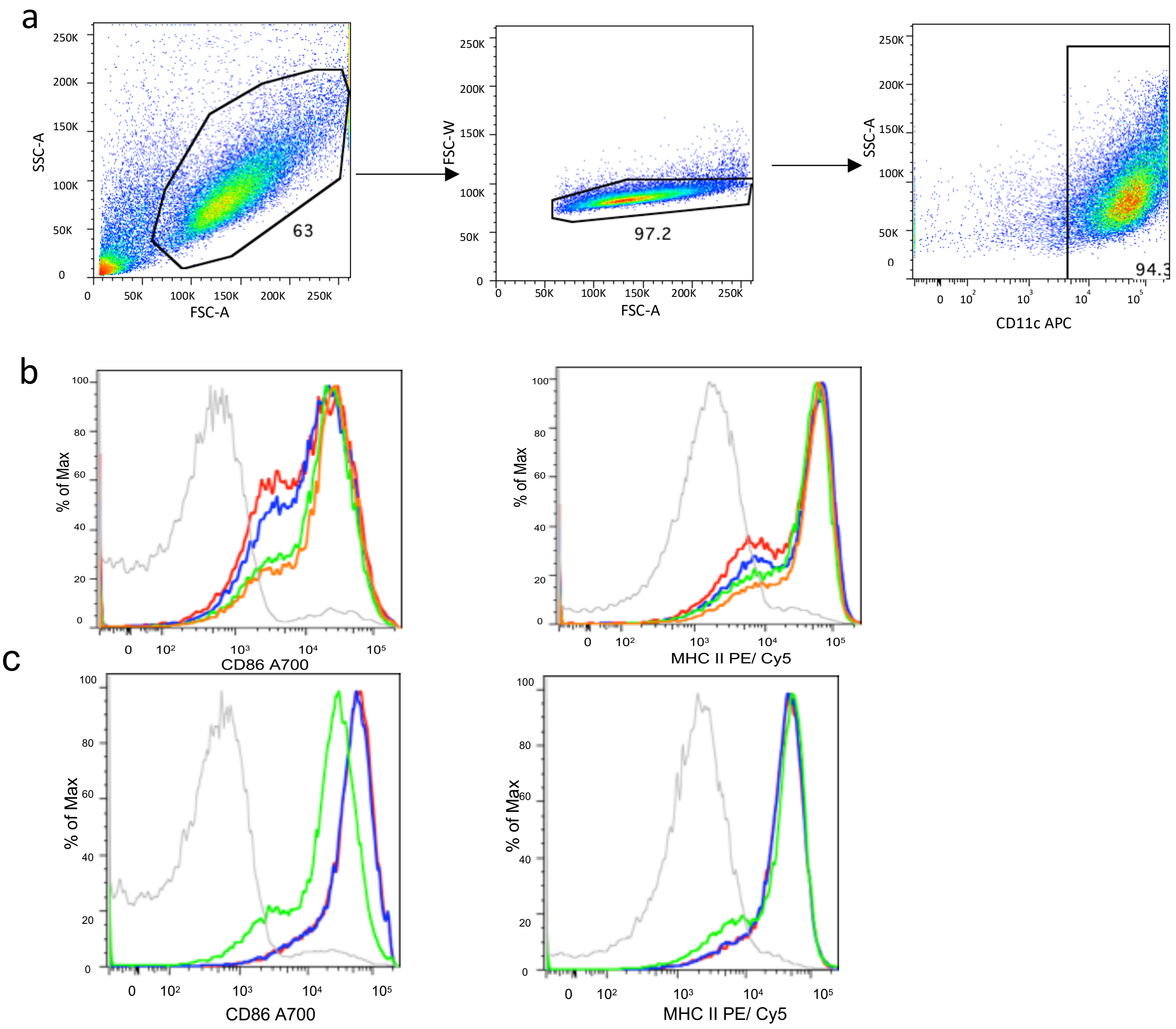

Supplementary Figure 12-Gating strategy for Supplementary Figure 9. a Strategy to define CD11c+ BMDCs that were used in the experiments for Supplementary Figure 9 **b-e. b** relates to Supplementary Figure 9 **b-c**, overlay plots show expression of CD86 and MHC II on CD11c+ BMDC, grey line: fluorescence minus control, red line: media, blue line: 10 μg/ml p43, green line: 50 μg/ml p43, orange line: 100 μg/ml p43. **c** relates to Supplementary Figure 9 **d-e** overlay plots show expression of CD86 and MHC II on CD11c+ BMDC, grey line: fluorescence minus control, red line: LPS alone, blue line: LPS ± 50 μg/ml p43, green line: LPS ± 25 μg/ml SEA.

Supplementary Table 2 for PDB ID 6QIX

	p43
Wavelength	0.92
Resolution range	57.34 - 1.65 (1.709 - 1.65)
Space group	P 1 21 1
Unit cell	47.6817 164.834 61.1689 90 110.37 90
Total reflections	899700 (86471)
Unique reflections	103460 (9969)
Multiplicity	8.7 (8.7)
Completeness (%)	97.72 (94.26)
Mean I/sigma(I)	9.57 (1.06)
Wilson B-factor	20.12
R-merge	0.1267 (1.953)
R-meas	0.1347 (2.078)
R-pim	0.04516 (0.7)
CC1/2	0.998 (0.528)
CC*	1 (0.831)
Reflections used in refinement	103433 (9962)
Reflections used for R-free	5140 (540)
R-work	0.1636 (0.2770)
R-free	0.1963 (0.3293)
CC(work)	0.964 (0.813)
CC(free)	0.961 (0.727)
Number of non-hydrogen atoms	6219
macromolecules	5610
ligands	140
solvent	469
Protein residues	694
RMS(bonds)	0.013
RMS(angles)	1.2
Ramachandran favored (%)	98.26
Ramachandran allowed (%)	1.16
Ramachandran outliers (%)	0.58
Rotamer outliers (%)	0.31
Clashscore	6.24
Average B-factor	33.14
macromolecules	32.54
ligands	51.77
solvent	34.74

Supplementary Table 2-Data collection and refinement statistics. Statistics for the highest resolution shell are shown in parentheses.

Methods

SDS PAGE of E/S

RPMI plus 500 IU/ml of penicillin and 500 μg/ml of streptomycin was used to culture adult *T. muris* and obtain E/S proteins. 1ml of culture medium was centrifuged at 19,000g to pellet eggs and then 10 μl was run on a NuPAGE 4-12% Bis-Tris gel (Life Sciences) at 200V using NuPAGE sample buffer and 0.05% v/v 2- mercaptoethanol and NuPAGE MES SDS running buffer. The sample was denatured at 95°C for 5 minutes before loading on to a gel with protein standards, LC5800 (Life Sciences). The gel was then stained for 1 hour using Instant Blue (Expedeon) and destained using distilled water. The gel was photographed using a digipad (Medline Scientific).

Multi angle light scattering

Multi-angle light scattering (MALS) was used to test the size, purity and oligomeric state of E/S and native p43. The proteins were passed down a Superdex 200 24/300 gel filtration column (GE healthcare) at a rate of 0.75 ml/minute in PBS buffer, pH7.4 using a NGC FPLC (Bio-Rad). The eluate passed through a Wyatt Helios 2 18-angle light scattering instrument and a rEX differential refractive index detector for accurate concentration detection using a *dn*/*dc* value of 0.185 ml/g. The resulting scattering peaks were analyzed using ASTRA version 6.1 to determine the average mass across the eluted peaks.

Tandem mass spectrometry

Mucus samples were reduced for 3 hours at 37°C in 50 mM dithiothreitol (DTT; Melford Labs) and alkylated with 100mM idoactemide for 15 minutes at room temperature in the dark. Samples were spun in Vivaspin (Sartorius) 3kDa Molecular Weight cut off (MWCO) columns for 10 minutes at 14,000g for buffer exchange into 100 mM ammonium bicarbonate. 5 μg of sequencing grade modified trypsin (Promega) was added to samples and incubated at 37 °C overnight. Centrifugation in Vivaspin 10kDa MWCO columns was carried out on the

digested product, thereby retaining high molecular weight glycopeptides within the filter and tryptic peptides in the flow through. The peptides were acidified to pH 2 using 0.1% (v/v) formic acid, and recovered free of salt by reverse phase chromatography using a C-18 ZipTip (Millipore, Durham, UK). Zip-tips were pre-washed with 4 cycles 50% (V/V) acetonitrile, followed by 8 cycles 0.1 % (v/v) formic acid and loaded with the sample. The sample was passed through the resin tip repeatedly before washing with 0.1% (v/v) formic acid. Peptides were eluted in 50% (v/v) acetonitrile, dried in a vacuum centrifuge for 10 minutes and re suspended in 10 μl 0.1 % (v/v) formic acid / 50 % (v/v) acetonitrile.

Samples were analyzed by tandem mass spectrometry (MS/MS) using a NanoAcuity LC coupled to a LTQ Velos mass spectrometer. Results were searched using Mascot (Matrix Science UK) and searched against the *T. muris* proteome, version 2.1 (Sanger Centre 2013) and murine database (acquired from UniProt_Murine). The following parameters were used for Mascot analysis: fixed modifications of carbamidomethyl-cysteine, variable modifications of methionine oxidation, peptide tolerance of 1.2Da, MS/MS tolerance of 0.6 Da, and peptide charge of 2+ and 3+. Following mascot analysis the resulting data were further analyzed using Scaffold (Proteome Software, Portland, OR) (version 4) using the following parameters: protein probability threshold of 80%, peptide probability threshold of 50% and minimum number of peptides 3.

L2, L3, L4 and adult *T. muris*

SCID mice were infected with 200 *T. muris* eggs and 2 mice were culled at day 14, day 21 and day 35 p.i. The cecum and proximal colon were flushed through with PBS, 0.9% NaCl. The cecum and colon were incubated with 10 ml of PBS, NaCl at 37 °C for 2 hours with frequent shaking. Individual larvae of various stages were picked out and washed in PBS. They were frozen at -80°C until processed. At day 35 p.i., males and females were separated. Embryonated eggs are routinely kept in the fridge in the laboratory.

Real Time, semi-quantitative PCR

RNA was extracted from different life cycle stages by maceration in Trizol (Life Technologies) with a FastPrep and lysing matrix D (MP Biomedical). 2 µg of RNA was used to synthesise RNA using GoScript reverse transcriptase (Promega) and a poly dT primer. Reverse transcriptase, Real Time, semi quantitative PCR was carried out using SensiFAST Sybr Hi-ROX (Bioline) and a StepOne Plus thermocycler (Applied Biosystems). 18S was used as a housekeeping gene and fold changes were calculated using the ΔΔCt method. The mean Ct of 3 technical replicates was used for each separate biological replicate (a pool of worms from an individual mouse). ΔΔCts were then calculated for each biological replicate (n=3) and the SEM was then calculated. A reverse transcriptase negative control was also included. Primer sequences, 18S Forward 5' GTTTACGGTGACGAGGCAAT 3' and Reverse 5' TCACAACTAGGGGCGGTATC 3 and for 43 Forward 5' CAAAAAGCACTGCATCGAGA 3' and Reverse 5' GTCGTAGACGTGGCTGACAA 3'.

Culturing *T. muris* with sodium azide

10 female and 15 male adult *T. muris* worms were cultured overnight in RPMI plus 500 IU/ml penicillin, 500 µg/ml streptomycin or with 10mM or 100mM sodium azide in a 24 tissue culture plate. 10 µl of culture medium from each condition was run on SDS PAGE as previously described.

Biotinylated Glycosaminoglycan (GAG) ELISAs

ELISA plates were coated with 5 µg/ml of either p43 or r43 in carbonate, bicarbonate buffer pH 9.6 and incubated at 4°C overnight. The next day plates were washed in PBS Tween 20, 0.05% and excess antigen binding sites were blocked with PBS, 1% FCS for 1 hour at room temperature. After washing again, the plates were incubated in a range of biotinylated HS or chondroitin 4 sulfate for 1 hour, washed and incubated in streptavidin peroxidase and, after a

final wash, developed in 3,3',5,5'-Tetramethylbenzidine (TMB, Becton Dickinson Opt EIA™ substrate, Catalog number, 555214). ELISA substrate. Plates were read on a VersaMax (Molecular Devices) and data analyzed by SoftMax Pro v 6.4.2

Immunostaining of bone marrow derived dendritic cells

Bone marrow was collected from femurs and tibias of male BALB/c mice. Red blood cells were lysed with Ack buffer (150mM ammonium chloride (NH_4Cl), 10mM potassium carbonate $(KCO)_3$, 0.1 mM Na_2EDTA) and the remaining cells cultured at $2x10^5$ cells/ml for 7 days in RPMI 1640 plus 100 IU/ml penicillin and 100 μg/ml streptomycin with 50 μM β-mercaptoethanol (Sigma), 10% FCS (Gibco) and 40 ng/ml mouse GM-CSF (eBiosciences). Dendritic cells (DCs) were collected from culture plates and re-plated at $1x10^6$ cells/ml onto poly-D lysine (Sigma-Aldrich UK) coated coverslips for 24h. Cells were then pulsed with 25 μg/ml Cy3 (GE Healthcare) labeled p43 for 1hour, washed and fresh media added for 2 hours before being washed in PBS and fixed with paraformaldehyde (Sigma) for 15 minutes. DCs were permeabilized with 0.1% Triton X-100 (Sigma), blocked with goat serum and stained with rat anti-mouse H2M (BD Biosciences) at 1/1000 dilution with a goat anti-rat-AlexaFluor 488 secondary (Invitrogen) before being mounted in Mowiol® 4-88 mounting medium with DAPI (Sigma-Aldrich UK). Images were acquired on an Olympus IX83 inverted microscope using Green Lumencor LED excitation, a 60x/ 1.42 Plan Apo objective and the Sedat filter set (Chroma 89000). The images were collected using a R6 (Qimaging) CCD camera with a Z optical spacing of 0.2μm. Raw images were then deconvolved using the Huygens Pro software (SVI) and maximum intensity projections of these deconvolved images obtained.

Bone marrow derived dendritic cells for flow cytometry

Bone marrow was collected from femurs of C57BL/6 mice. Red blood cells were lysed and the remaining cells cultured at $2x10^5$ cells/ml for 7 days in RPMI 1640 with 2mM L-glutamine, penicillin/streptomycin (all Sigma), 10% FCS (Gibco) and 20ng/ml mouse

Granulocyte Mast Cell-Colony Stimulating Factor (GM-CSF) (Biolegend). At day 3, a further 10ml of media containing 20 ng/ml of GMCS-F was added. At day 6, 8 and 9 culture supernatant was removed and replaced with 10ml of fresh culture medium containing 20ng/ml GM-CSF. On day 10 GMDCs were collected from culture plates and cultured at 2 x 10^6 cells in triplicate for 18 hours with 10, 50 or 100 µg/ml of p43. In experiments using lipopolysaccharide (LPS), 250 ng/ml of LPS was used and 25 µg/ml of *S. mansoni* egg antigen (SEA). CD11C APC+ cells were stained for MHC II PE.Cy5 and CD86.A700. Samples were acquired using a 5 laser Fortessa with BD FACSDiva software and analyzed with FlowJo software (v9, Tree Star).

Supplementary References

1 Blaxter, M. & Koutsovoulos, G. The evolution of parasitism in Nematoda. *Parasitology* **142 Suppl 1**, S26-39, doi:10.1017/S0031182014000791 (2015).

2 Henikoff, S. & Henikoff, J. G. Amino acid substitution matrices from protein blocks. *Proc Natl Acad Sci U S A* **89**, 10915-10919 (1992).

3 Korhonen, P. K. *et al.* Phylogenomic and biogeographic reconstruction of the Trichinella complex. *Nat Commun* **7**, 10513, doi:10.1038/ncomms10513 (2016).

WORLDWIDE PDB PROTEIN DATA BANK

Full wwPDB X-ray Structure Validation Report (i)

Jan 22, 2019 – 12:06 PM GMT

PDB ID	:	6QIX
Title	:	The crystal structure of Trichuris muris P43
Deposited on	:	2019-01-21
Resolution	:	1.65 Å(reported)

This is a Full wwPDB X-ray Structure Validation Report.

This report is produced by the wwPDB biocuration pipeline after annotation of the structure.

We welcome your comments at *validation@mail.wwpdb.org*
A user guide is available at
https://www.wwpdb.org/validation/2017/XrayValidationReportHelp
with specific help available everywhere you see the (i) symbol.

CONFIDENTIAL VALIDATION REPORT

The following versions of software and data (see references (i)) were used in the production of this report:

MolProbity	:	4.02b-467
Mogul	:	1.7.3 (157068), CSD as539be (2018)
Xtriage (Phenix)	:	1.13
EDS	:	FAILED
Percentile statistics	:	20171227.v01 (using entries in the PDB archive December 27th 2017)
Ideal geometry (proteins)	:	Engh & Huber (2001)
Ideal geometry (DNA, RNA)	:	Parkinson et al. (1996)
Validation Pipeline (wwPDB-VP)	:	rb-20031633

1 Overall quality at a glance (i)

The following experimental techniques were used to determine the structure:
X-RAY DIFFRACTION

The reported resolution of this entry is 1.65 Å.

Percentile scores (ranging between 0-100) for global validation metrics of the entry are shown in the following graphic. The table shows the number of entries on which the scores are based.

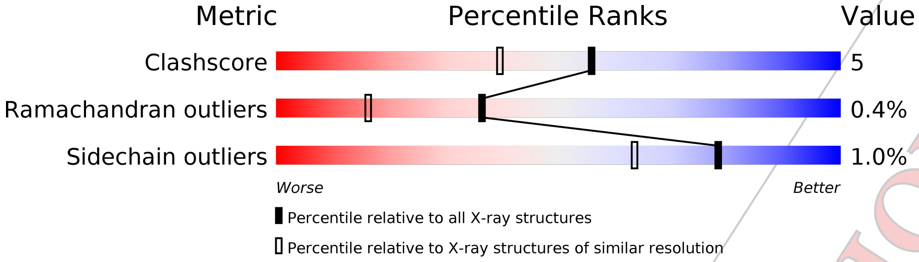

Metric	Whole archive (#Entries)	Similar resolution (#Entries, resolution range(Å))
Clashscore	122126	1616 (1.66-1.66)
Ramachandran outliers	120053	1584 (1.66-1.66)
Sidechain outliers	120020	1584 (1.66-1.66)

The table below summarises the geometric issues observed across the polymeric chains and their fit to the electron density. The red, orange, yellow and green segments on the lower bar indicate the fraction of residues that contain outliers for >=3, 2, 1 and 0 types of geometric quality criteria. A grey segment represents the fraction of residues that are not modelled. The numeric value for each fraction is indicated below the corresponding segment, with a dot representing fractions <=5%

Note EDS failed to run properly.

Mol	Chain	Length	Quality of chain
1	A	394	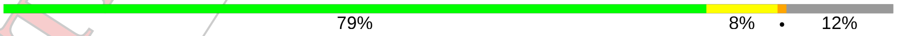 79% 8% • 12%
1	B	394	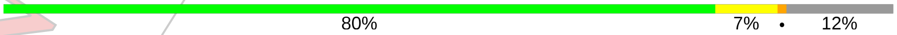 80% 7% • 12%

CONFIDENTIAL VALIDATION REPORT

2 Entry composition (i)

There are 7 unique types of molecules in this entry. The entry contains 12036 atoms, of which 5527 are hydrogens and 0 are deuteriums.

In the tables below, the ZeroOcc column contains the number of atoms modelled with zero occupancy, the AltConf column contains the number of residues with at least one atom in alternate conformation and the Trace column contains the number of residues modelled with at most 2 atoms.

- Molecule 1 is a protein called Uncharacterized protein.

Mol	Chain	Residues	Atoms						ZeroOcc	AltConf	Trace
			Total	C	H	N	O	S			
1	A	347	5494	1776	2669	498	503	48	0	9	0
1	B	347	5443	1762	2636	493	505	47	0	6	0

- Molecule 2 is N-ACETYL-D-GLUCOSAMINE (three-letter code: NAG) (formula: $C_8H_{15}NO_6$).

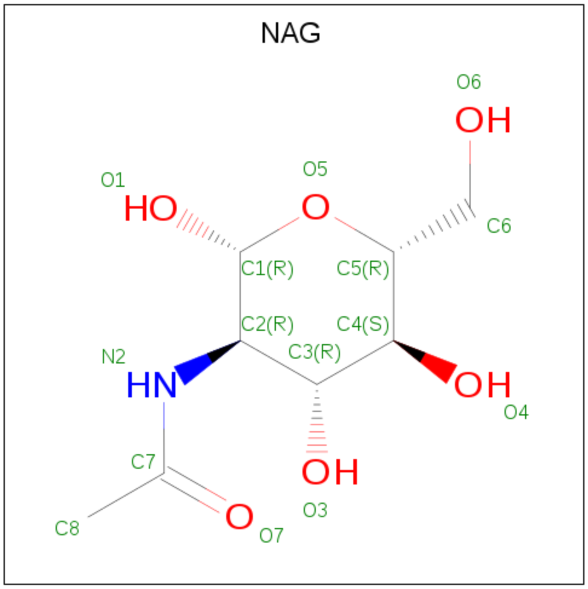

Mol	Chain	Residues	Atoms					ZeroOcc	AltConf
			Total	C	H	N	O		
2	A	1	28	8	14	1	5	0	0
2	A	1	28	8	14	1	5	0	0
2	B	1	28	8	14	1	5	0	0
2	B	1	28	8	14	1	5	0	0

CONFIDENTIAL VALIDATION REPORT

- Molecule 3 is ACETATE ION (three-letter code: ACT) (formula: $C_2H_3O_2$).

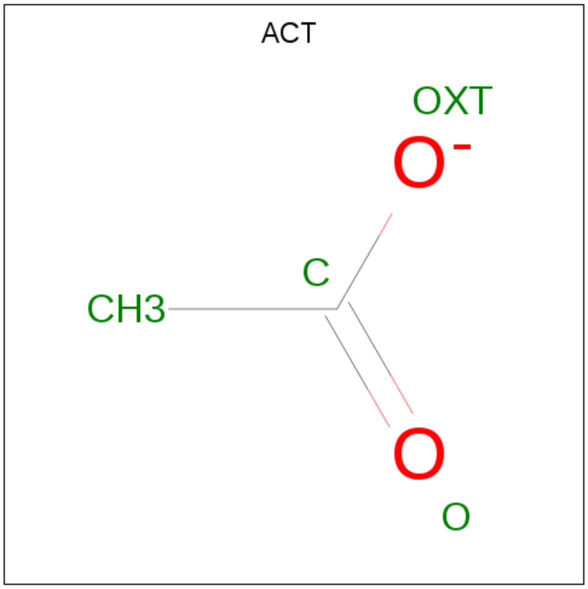

Mol	Chain	Residues	Atoms	ZeroOcc	AltConf
3	A	1	Total C H O<br>7 2 3 2	0	0
3	A	1	Total C H O<br>7 2 3 2	0	0
3	A	1	Total C H O<br>7 2 3 2	0	0
3	B	1	Total C H O<br>7 2 3 2	0	0

- Molecule 4 is HEXAETHYLENE GLYCOL (three-letter code: P6G) (formula: $C_{12}H_{26}O_7$).

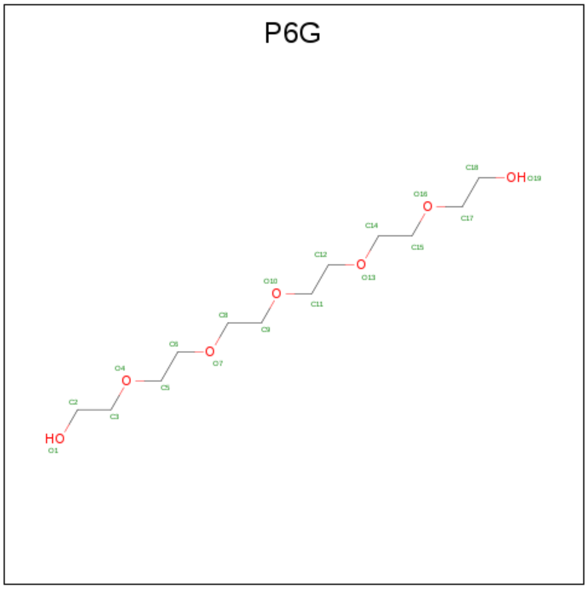

Mol	Chain	Residues	Atoms	ZeroOcc	AltConf
4	A	1	Total C H O<br>45 12 26 7	0	0
4	A	1	Total C H O<br>45 12 26 7	0	0
4	B	1	Total C H O<br>45 12 26 7	0	0
4	B	1	Total C H O<br>45 12 26 7	0	0

- Molecule 5 is CALCIUM ION (three-letter code: CA) (formula: Ca).

Mol	Chain	Residues	Atoms	ZeroOcc	AltConf
5	A	1	Total Ca<br>1 1	0	0

- Molecule 6 is DI(HYDROXYETHYL)ETHER (three-letter code: PEG) (formula: $C_4H_{10}O_3$).

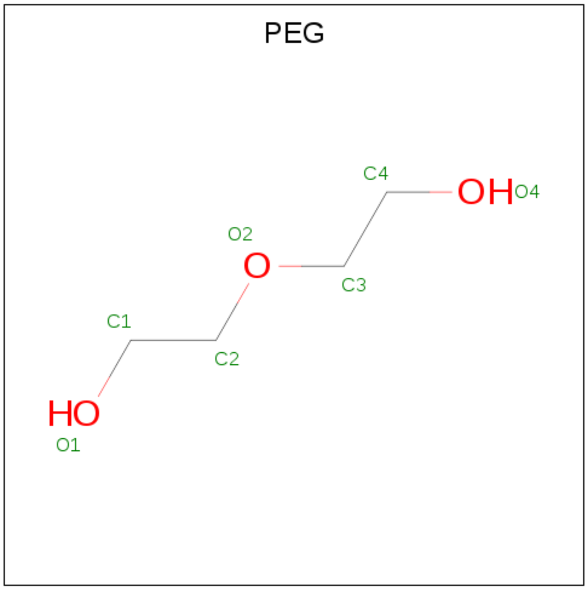

Mol	Chain	Residues	Atoms	ZeroOcc	AltConf
6	A	1	Total C H O<br>17 4 10 3	0	0
6	A	1	Total C H O<br>17 4 10 3	0	0
6	B	1	Total C H O<br>17 4 10 3	0	0
6	B	1	Total C H O<br>17 4 10 3	0	0
6	B	1	Total C H O<br>17 4 10 3	0	0

CONFIDENTIAL VALIDATION REPORT

- Molecule 7 is water.

Mol	Chain	Residues	Atoms	ZeroOcc	AltConf
7	A	333	Total O<br>333 333	0	0
7	B	360	Total O<br>360 360	0	0

CONFIDENTIAL VALIDATION REPORT

3 Residue-property plots (i)

These plots are drawn for all protein, RNA and DNA chains in the entry. The first graphic for a chain summarises the proportions of the various outlier classes displayed in the second graphic. The second graphic shows the sequence view annotated by issues in geometry. Residues are color-coded according to the number of geometric quality criteria for which they contain at least one outlier: green = 0, yellow = 1, orange = 2 and red = 3 or more. Stretches of 2 or more consecutive residues without any outlier are shown as a green connector. Residues present in the sample, but not in the model, are shown in grey.

Note EDS failed to run properly.

- Molecule 1: Uncharacterized protein

Chain A:

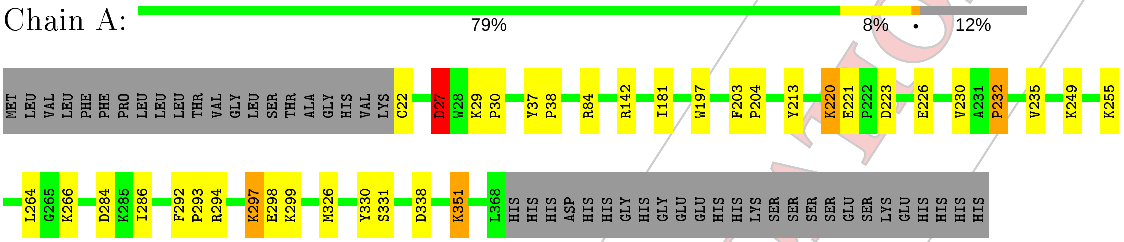

- Molecule 1: Uncharacterized protein

Chain B:

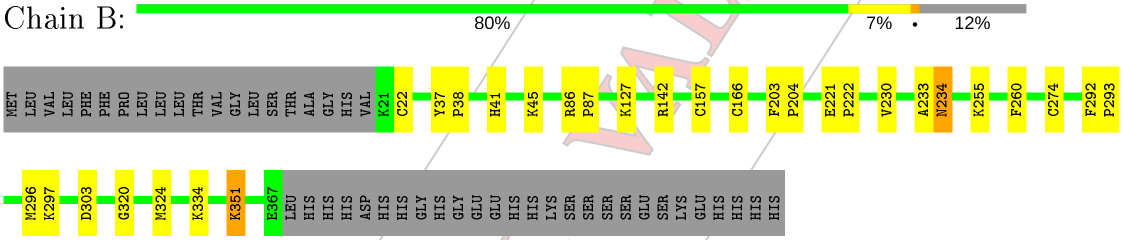

CONFIDENTIAL VALIDATION REPORT

4 Data and refinement statistics (i)

EDS failed to run properly - this section is therefore incomplete.

Property	Value	Source
Space group	P 1 21 1	Depositor
Cell constants<br>a, b, c, α, β, γ	47.68Å 164.83Å 61.17Å<br>90.00° 110.37° 90.00°	Depositor
Resolution (Å)	57.34 – 1.65	Depositor
% Data completeness<br>(in resolution range)	97.7 (57.34-1.65)	Depositor
R_{merge}	0.13	Depositor
R_{sym}	(Not available)	Depositor
$< I/\sigma(I) >$ [1]	0.88 (at 1.65Å)	Xtriage
Refinement program	PHENIX dev_3304	Depositor
R, R_{free}	0.150 , 0.189	Depositor
Wilson B-factor (Å^2)	17.3	Xtriage
Anisotropy	0.521	Xtriage
L-test for twinning[2]	$< \|L\| > = 0.49$, $< L^2 > = 0.32$	Xtriage
Estimated twinning fraction	0.043 for h,-k,-h-l	Xtriage
Total number of atoms	12036	wwPDB-VP
Average B, all atoms (Å^2)	35.0	wwPDB-VP

Xtriage's analysis on translational NCS is as follows: *The largest off-origin peak in the Patterson function is 5.10% of the height of the origin peak. No significant pseudotranslation is detected.*

[1] Intensities estimated from amplitudes.

[2] Theoretical values of $< |L| >$, $< L^2 >$ for acentric reflections are 0.5, 0.333 respectively for untwinned datasets, and 0.375, 0.2 for perfectly twinned datasets.

CONFIDENTIAL VALIDATION REPORT

5 Model quality (i)

5.1 Standard geometry (i)

Bond lengths and bond angles in the following residue types are not validated in this section: CA, PEG, NAG, P6G, ACT

The Z score for a bond length (or angle) is the number of standard deviations the observed value is removed from the expected value. A bond length (or angle) with $|Z| > 5$ is considered an outlier worth inspection. RMSZ is the root-mean-square of all Z scores of the bond lengths (or angles).

Mol	Chain	Bond lengths		Bond angles	
		RMSZ	$\#\|Z\| > 5$	RMSZ	$\#\|Z\| > 5$
1	A	0.61	0/2928	0.65	1/3954 (0.0%)
1	B	0.61	0/2901	0.71	2/3920 (0.1%)
All	All	0.61	0/5829	0.68	3/7874 (0.0%)

Chiral center outliers are detected by calculating the chiral volume of a chiral center and verifying if the center is modelled as a planar moiety or with the opposite hand.A planarity outlier is detected by checking planarity of atoms in a peptide group, atoms in a mainchain group or atoms of a sidechain that are expected to be planar.

Mol	Chain	#Chirality outliers	#Planarity outliers
1	A	0	1

There are no bond length outliers.

All (3) bond angle outliers are listed below:

Mol	Chain	Res	Type	Atoms	Z	Observed(°)	Ideal(°)
1	B	324	MET	CG-SD-CE	10.74	117.38	100.20
1	A	264	LEU	CA-CB-CG	5.39	127.69	115.30
1	B	142	ARG	NE-CZ-NH2	-5.11	117.75	120.30

There are no chirality outliers.

All (1) planarity outliers are listed below:

Mol	Chain	Res	Type	Group
1	A	27	ASP	Peptide

CONFIDENTIAL VALIDATION REPORT

5.2 Too-close contacts (i)

In the following table, the Non-H and H(model) columns list the number of non-hydrogen atoms and hydrogen atoms in the chain respectively. The H(added) column lists the number of hydrogen atoms added and optimized by MolProbity. The Clashes column lists the number of clashes within the asymmetric unit, whereas Symm-Clashes lists symmetry related clashes.

Mol	Chain	Non-H	H(model)	H(added)	Clashes	Symm-Clashes
1	A	2825	2669	2653	42	0
1	B	2807	2636	2633	19	0
2	A	28	28	26	4	0
2	B	28	28	26	0	0
3	A	12	9	9	0	0
3	B	4	3	3	0	0
4	A	38	52	52	4	0
4	B	38	52	52	0	0
5	A	1	0	0	0	0
6	A	14	20	20	3	0
6	B	21	30	30	0	0
7	A	333	0	0	7	1
7	B	360	0	0	4	2
All	All	6509	5527	5504	62	2

The all-atom clashscore is defined as the number of clashes found per 1000 atoms (including hydrogen atoms). The all-atom clashscore for this structure is 5.

All (62) close contacts within the same asymmetric unit are listed below, sorted by their clash magnitude.

Atom-1	Atom-2	Interatomic distance (Å)	Clash overlap (Å)
1:A:297:LYS:NZ	7:A:501:HOH:O	1.63	1.23
1:B:303[B]:ASP:OD2	7:B:501:HOH:O	1.65	1.12
1:A:286:ILE:HG21	2:A:406:NAG:H83	1.52	0.89
1:B:22:CYS:SG	7:B:809:HOH:O	2.31	0.89
1:A:351:LYS:HE2	1:A:351:LYS:H	1.46	0.80
1:A:232:PRO:HG3	2:A:406:NAG:H81	1.64	0.79
1:A:221:GLU:OE2	7:A:502:HOH:O	2.00	0.79
1:B:37:TYR:HB3	1:B:38:PRO:HA	1.75	0.68
1:B:41:HIS:NE2	1:B:45:LYS:HE2	2.10	0.66
1:A:37:TYR:HB3	1:A:38:PRO:HA	1.76	0.66
1:A:351:LYS:HE2	1:A:351:LYS:N	2.17	0.59
1:B:127:LYS:NZ	7:B:502:HOH:O	2.36	0.58
1:A:230:VAL:HG11	1:A:299:LYS:HG3	1.84	0.58
1:A:330:TYR:CG	6:A:409:PEG:H12	2.39	0.58

Continued on next page...

CONFIDENTIAL VALIDATION REPORT

Continued from previous page...

Atom-1	Atom-2	Interatomic distance (Å)	Clash overlap (Å)
1:B:233:ALA:O	1:B:234:ASN:CB	2.51	0.58
1:A:293:PRO:O	4:A:403:P6G:H171	2.06	0.56
1:A:331:SER:H	4:A:403:P6G:H181	1.71	0.56
1:A:330:TYR:CD1	6:A:409:PEG:H12	2.41	0.55
1:A:181[A]:ILE:HD11	7:A:831:HOH:O	2.05	0.55
1:A:298:GLU:OE1	7:A:503:HOH:O	2.18	0.54
2:A:406:NAG:H3	2:A:406:NAG:O7	2.08	0.52
1:A:232:PRO:HD2	1:A:235:VAL:HG21	1.91	0.52
1:A:286:ILE:CG2	2:A:406:NAG:H83	2.34	0.52
1:A:213:TYR:OH	1:A:220:LYS:HE2	2.09	0.52
1:B:334:LYS:NZ	1:B:351:LYS:HA	2.25	0.52
1:A:255:LYS:NZ	7:A:510:HOH:O	2.42	0.52
1:B:233:ALA:O	1:B:234:ASN:HB3	2.12	0.50
1:A:29:LYS:HB3	1:A:30:PRO:HD2	1.94	0.50
1:B:86:ARG:HB2	1:B:87:PRO:HD2	1.94	0.50
1:A:330:TYR:HA	4:A:403:P6G:H181	1.94	0.48
1:A:27:ASP:HA	1:A:84:ARG:CB	2.42	0.48
1:B:157:CYS:SG	1:B:166[B]:CYS:SG	3.06	0.48
1:A:142:ARG:NH1	1:A:266:LYS:O	2.43	0.48
1:A:297:LYS:HE3	1:A:330:TYR:HD1	1.78	0.48
1:A:220:LYS:HE3	1:A:226:GLU:HB2	1.96	0.47
1:A:294:ARG:HD3	1:A:298:GLU:OE2	2.14	0.47
1:A:326:MET:SD	6:A:409:PEG:H32	2.55	0.47
1:B:292:PHE:CG	1:B:293:PRO:HD3	2.50	0.47
1:A:181[B]:ILE:HD12	1:A:181[B]:ILE:C	2.35	0.47
1:A:22:CYS:N	7:A:516:HOH:O	2.48	0.46
1:A:181[A]:ILE:CD1	7:A:831:HOH:O	2.64	0.46
1:B:230:VAL:HG21	1:B:296:MET:O	2.16	0.45
1:A:27:ASP:HA	1:A:84:ARG:HB3	1.98	0.45
1:A:181[B]:ILE:HD12	1:A:181[B]:ILE:O	2.16	0.45
1:A:230:VAL:HG11	1:A:299:LYS:CG	2.48	0.44
1:B:221:GLU:OE2	1:B:255:LYS:HE3	2.17	0.43
1:A:293:PRO:CB	1:A:331:SER:OG	2.66	0.43
1:B:351:LYS:HB2	1:B:351:LYS:HE2	1.86	0.43
1:B:260:PHE:CG	1:B:320:GLY:HA3	2.55	0.42
1:A:338[A]:ASP:OD2	1:A:338[A]:ASP:N	2.48	0.42
1:A:293:PRO:HB3	1:A:331:SER:OG	2.20	0.41
1:B:203:PHE:HB3	1:B:204:PRO:HA	2.03	0.41
1:B:221:GLU:HG3	1:B:222:PRO:HD2	2.01	0.41
1:A:197:TRP:CE2	1:A:249:LYS:HE3	2.56	0.41
1:A:203:PHE:HB3	1:A:204:PRO:HA	2.03	0.41

Continued on next page...

CONFIDENTIAL VALIDATION REPORT

Continued from previous page...

Atom-1	Atom-2	Interatomic distance (Å)	Clash overlap (Å)
1:B:297:LYS:NZ	7:B:515:HOH:O	2.53	0.41
1:B:234:ASN:ND2	1:B:234:ASN:O	2.54	0.41
1:A:292:PHE:N	1:A:293:PRO:CD	2.83	0.41
1:A:331:SER:N	4:A:403:P6G:H181	2.33	0.41
1:A:232:PRO:HB2	1:A:235:VAL:HG23	2.02	0.40
1:A:221:GLU:HG2	1:A:223:ASP:OD2	2.21	0.40

All (2) symmetry-related close contacts are listed below. The label for Atom-2 includes the symmetry operator and encoded unit-cell translations to be applied.

Atom-1	Atom-2	Interatomic distance (Å)	Clash overlap (Å)
7:A:687:HOH:O	7:B:817:HOH:O[2_445]	2.12	0.08
7:B:614:HOH:O	7:B:789:HOH:O[1_655]	2.18	0.02

5.3 Torsion angles (i)

5.3.1 Protein backbone (i)

In the following table, the Percentiles column shows the percent Ramachandran outliers of the chain as a percentile score with respect to all X-ray entries followed by that with respect to entries of similar resolution.

The Analysed column shows the number of residues for which the backbone conformation was analysed, and the total number of residues.

Mol	Chain	Analysed	Favoured	Allowed	Outliers	Percentiles	
1	A	354/394 (90%)	346 (98%)	6 (2%)	2 (1%)	27	8
1	B	351/394 (89%)	345 (98%)	5 (1%)	1 (0%)	43	22
All	All	705/788 (90%)	691 (98%)	11 (2%)	3 (0%)	36	16

All (3) Ramachandran outliers are listed below:

Mol	Chain	Res	Type
1	B	234	ASN
1	A	27	ASP
1	A	232	PRO

CONFIDENTIAL VALIDATION REPORT

5.3.2 Protein sidechains (i)

In the following table, the Percentiles column shows the percent sidechain outliers of the chain as a percentile score with respect to all X-ray entries followed by that with respect to entries of similar resolution.

The Analysed column shows the number of residues for which the sidechain conformation was analysed, and the total number of residues.

Mol	Chain	Analysed	Rotameric	Outliers	Percentiles	
1	A	321/354 (91%)	317 (99%)	4 (1%)	74	55
1	B	318/354 (90%)	316 (99%)	2 (1%)	87	78
All	All	639/708 (90%)	633 (99%)	6 (1%)	78	67

All (6) residues with a non-rotameric sidechain are listed below:

Mol	Chain	Res	Type
1	A	27	ASP
1	A	220	LYS
1	A	297	LYS
1	A	351	LYS
1	B	274	CYS
1	B	351	LYS

Some sidechains can be flipped to improve hydrogen bonding and reduce clashes. All (1) such sidechains are listed below:

Mol	Chain	Res	Type
1	B	234	ASN

5.3.3 RNA (i)

There are no RNA molecules in this entry.

5.4 Non-standard residues in protein, DNA, RNA chains (i)

There are no non-standard protein/DNA/RNA residues in this entry.

5.5 Carbohydrates (i)

There are no carbohydrates in this entry.

CONFIDENTIAL VALIDATION REPORT

5.6 Ligand geometry (i)

Of 18 ligands modelled in this entry, 1 is monoatomic - leaving 17 for Mogul analysis.

In the following table, the Counts columns list the number of bonds (or angles) for which Mogul statistics could be retrieved, the number of bonds (or angles) that are observed in the model and the number of bonds (or angles) that are defined in the Chemical Component Dictionary. The Link column lists molecule types, if any, to which the group is linked. The Z score for a bond length (or angle) is the number of standard deviations the observed value is removed from the expected value. A bond length (or angle) with $|Z| > 2$ is considered an outlier worth inspection. RMSZ is the root-mean-square of all Z scores of the bond lengths (or angles).

Mol	Type	Chain	Res	Link	Bond lengths			Bond angles		
					Counts	RMSZ	$\#\|Z\| > 2$	Counts	RMSZ	$\#\|Z\| > 2$
2	NAG	A	401	1	14,14,15	0.52	0	17,19,21	0.59	0
3	ACT	A	402	-	1,3,3	4.75	1 (100%)	0,3,3	0.00	-
4	P6G	A	403	-	18,18,18	0.57	0	17,17,17	0.49	0
4	P6G	A	404	-	18,18,18	0.54	0	17,17,17	0.54	0
3	ACT	A	405	-	1,3,3	4.60	1 (100%)	0,3,3	0.00	-
2	NAG	A	406	1	14,14,15	0.59	1 (7%)	17,19,21	0.71	1 (5%)
6	PEG	A	408	-	6,6,6	0.49	0	5,5,5	0.38	0
6	PEG	A	409	-	6,6,6	0.52	0	5,5,5	0.84	0
3	ACT	A	410	-	1,3,3	6.57	1 (100%)	0,3,3	0.00	-
2	NAG	B	401	1	14,14,15	0.54	0	17,19,21	1.76	3 (17%)
4	P6G	B	402	-	18,18,18	0.54	0	17,17,17	0.57	0
4	P6G	B	403	-	18,18,18	0.54	0	17,17,17	0.57	0
2	NAG	B	404	1	14,14,15	0.86	1 (7%)	17,19,21	0.89	1 (5%)
6	PEG	B	405	-	6,6,6	0.48	0	5,5,5	0.42	0
6	PEG	B	406	-	6,6,6	0.57	0	5,5,5	0.67	0
6	PEG	B	407	-	6,6,6	0.51	0	5,5,5	0.47	0
3	ACT	B	408	-	1,3,3	7.55	1 (100%)	0,3,3	0.00	-

In the following table, the Chirals column lists the number of chiral outliers, the number of chiral centers analysed, the number of these observed in the model and the number defined in the Chemical Component Dictionary. Similar counts are reported in the Torsion and Rings columns. '-' means no outliers of that kind were identified.

Mol	Type	Chain	Res	Link	Chirals	Torsions	Rings
2	NAG	A	401	1	-	0/6/23/26	0/1/1/1
3	ACT	A	402	-	-	0/0/0/0	0/0/0/0
4	P6G	A	403	-	-	0/16/16/16	0/0/0/0
4	P6G	A	404	-	-	0/16/16/16	0/0/0/0
3	ACT	A	405	-	-	0/0/0/0	0/0/0/0
2	NAG	A	406	1	-	0/6/23/26	0/1/1/1

Continued on next page...

CONFIDENTIAL VALIDATION REPORT

Continued from previous page...

Mol	Type	Chain	Res	Link	Chirals	Torsions	Rings
6	PEG	A	408	-	-	0/4/4/4	0/0/0/0
6	PEG	A	409	-	-	0/4/4/4	0/0/0/0
3	ACT	A	410	-	-	0/0/0/0	0/0/0/0
2	NAG	B	401	1	-	0/6/23/26	0/1/1/1
4	P6G	B	402	-	-	0/16/16/16	0/0/0/0
4	P6G	B	403	-	-	0/16/16/16	0/0/0/0
2	NAG	B	404	1	-	0/6/23/26	0/1/1/1
6	PEG	B	405	-	-	0/4/4/4	0/0/0/0
6	PEG	B	406	-	-	0/4/4/4	0/0/0/0
6	PEG	B	407	-	-	0/4/4/4	0/0/0/0
3	ACT	B	408	-	-	0/0/0/0	0/0/0/0

All (6) bond length outliers are listed below:

Mol	Chain	Res	Type	Atoms	Z	Observed(Å)	Ideal(Å)
2	A	406	NAG	O5-C1	2.00	1.47	1.43
2	B	404	NAG	O5-C1	3.00	1.48	1.43
3	A	405	ACT	CH3-C	4.60	1.54	1.48
3	A	402	ACT	CH3-C	4.75	1.54	1.48
3	A	410	ACT	CH3-C	6.57	1.57	1.48
3	B	408	ACT	CH3-C	7.55	1.58	1.48

All (5) bond angle outliers are listed below:

Mol	Chain	Res	Type	Atoms	Z	Observed(^{o})	Ideal(^{o})
2	B	401	NAG	C1-C2-N2	2.04	113.97	110.49
2	B	401	NAG	C3-C4-C5	2.11	114.02	110.24
2	A	406	NAG	C1-O5-C5	2.16	115.16	112.19
2	B	404	NAG	C1-O5-C5	2.94	116.23	112.19
2	B	401	NAG	C1-O5-C5	5.22	119.37	112.19

There are no chirality outliers.

There are no torsion outliers.

There are no ring outliers.

3 monomers are involved in 11 short contacts:

Mol	Chain	Res	Type	Clashes	Symm-Clashes
4	A	403	P6G	4	0
2	A	406	NAG	4	0
6	A	409	PEG	3	0

CONFIDENTIAL VALIDATION REPORT

5.7 Other polymers (i)

There are no such residues in this entry.

5.8 Polymer linkage issues (i)

There are no chain breaks in this entry.

CONFIDENTIAL VALIDATION REPORT

6 Fit of model and data (i)

6.1 Protein, DNA and RNA chains (i)

EDS failed to run properly - this section is therefore empty.

6.2 Non-standard residues in protein, DNA, RNA chains (i)

EDS failed to run properly - this section is therefore empty.

6.3 Carbohydrates (i)

EDS failed to run properly - this section is therefore empty.

6.4 Ligands (i)

EDS failed to run properly - this section is therefore empty.

6.5 Other polymers (i)

EDS failed to run properly - this section is therefore empty.

CONFIDENTIAL VALIDATION REPORT
