# Supplementary material for: The major secreted protein of the whipworm parasite tethers to matrix and inhibits interleukin-13 function
Source: Nat Commun. 2019 May 28;10:2344. doi: 10.1038/s41467-019-09996-z (PMC6538607; doi:10.1038/s41467-019-09996-z)
Supplement: Supplementary file 3 — Source Data [file 41467_2019_9996_MOESM3_ESM.zip › source data/3b/P43_ED_FIG_6.html]

P43\_ED\_FIG\_6.html
